# Aurora B-INCENP Localization at Centromeres/Inner Kinetochores Is Required for Chromosome Bi-orientation in Budding Yeast

**DOI:** 10.1016/j.cub.2019.03.051

**Published:** 2019-05-06

**Authors:** Luis J. García-Rodríguez, Taciana Kasciukovic, Viola Denninger, Tomoyuki U. Tanaka

**Affiliations:** 1 Centre for Gene Regulation and Expression, School of Life Sciences, University of Dundee, Dow Street, Dundee DD1 5EH, UK

**Keywords:** Aurora B, Ipl1, INCENP, Sli15, survivin, Bir1, COMA, Mcm21, chromosome bi-orientation, kinetochore

## Abstract

For proper chromosome segregation in mitosis, sister kinetochores must interact with microtubules from opposite spindle poles (chromosome bi-orientation) [1, 2]. To promote bi-orientation, Aurora B kinase disrupts aberrant kinetochore-microtubule interactions [3, 4, 5, 6]. It has long been debated how Aurora B halts this action when bi-orientation is established and tension is applied across sister kinetochores. A popular explanation for it is that, upon bi-orientation, sister kinetochores are pulled in opposite directions, stretching the outer kinetochores [7, 8] and moving Aurora B substrates away from Aurora-B-localizing sites at centromeres (spatial separation model) [3, 5, 9]. This model predicts that Aurora B localization at centromeres is required for bi-orientation. However, this notion was challenged by the observation that Bir1 (yeast survivin), which recruits Ipl1-Sli15 (yeast Aurora B-INCENP) to centromeres, can become dispensable for bi-orientation [10]. This raised the possibility that Aurora B localization at centromeres is dispensable for bi-orientation. Alternatively, there might be a Bir1-independent mechanism for recruiting Ipl1-Sli15 to centromeres or inner kinetochores [5, 9]. Here, we show that the COMA inner kinetochore sub-complex physically interacts with Sli15, recruits Ipl1-Sli15 to the inner kinetochore, and promotes chromosome bi-orientation, independently of Bir1, in budding yeast. Moreover, using an engineered recruitment of Ipl1-Sli15 to the inner kinetochore when both Bir1 and COMA are defective, we show that localization of Ipl1-Sli15 at centromeres or inner kinetochores is required for bi-orientation. Our results give important insight into how Aurora B disrupts kinetochore-microtubule interaction in a tension-dependent manner to promote chromosome bi-orientation.

## Highlights

• The COMA inner kinetochore sub-complex facilitates chromosome bi-orientation
• COMA physically interacts with Sli15 and recruits Ipl1-Sli15 to the inner kinetochore
• This function of COMA is independent of Bir1 and its role supporting robust cohesion
• Localizing Ipl1-Sli15 at centromeres/inner kinetochores is crucial for bi-orientation

## Teaser

García-Rodríguez et al. show that the COMA inner kinetochore sub-complex physically interacts with Sli15, recruits Ipl1-Sli15 to the inner kinetochore, and promotes chromosome bi-orientation, independently of Bir1, in budding yeast. They conclude that localization of Ipl1-Sli15 at centromeres/inner kinetochores is required for bi-orientation.

## Results and Discussion

### COMA Facilitates Chromosome Bi-orientation, Independently of Bir1 and of Its Role in Supporting Robust Peri-centromere Cohesion

If there were a Bir1-independent mechanism of recruiting Ipl1-Sli15 to centromeres or inner kinetochores, regulators of such a mechanism might show negative genetic interaction with *bir1* mutants. In fact, both *bir1-17* mutant and a *sli15* mutant lacking its Bir1-binding domain showed a negative genetic interaction with *mcm21Δ* and *ctf19Δ* [10, 11]. Mcm21 and Ctf19 are non-essential components of the inner kinetochore sub-complex COMA (Ctf19-Okp1-Mcm21-Ame1) [12]. Another study showed that Sli15 localization at kinetochores was partially diminished in *ame1* mutants [13]. Thus, COMA may recruit Ipl1-Sli15 to inner kinetochores independently of Bir1 to promote bi-orientation and maintain cell viability.

**Figure 1:**
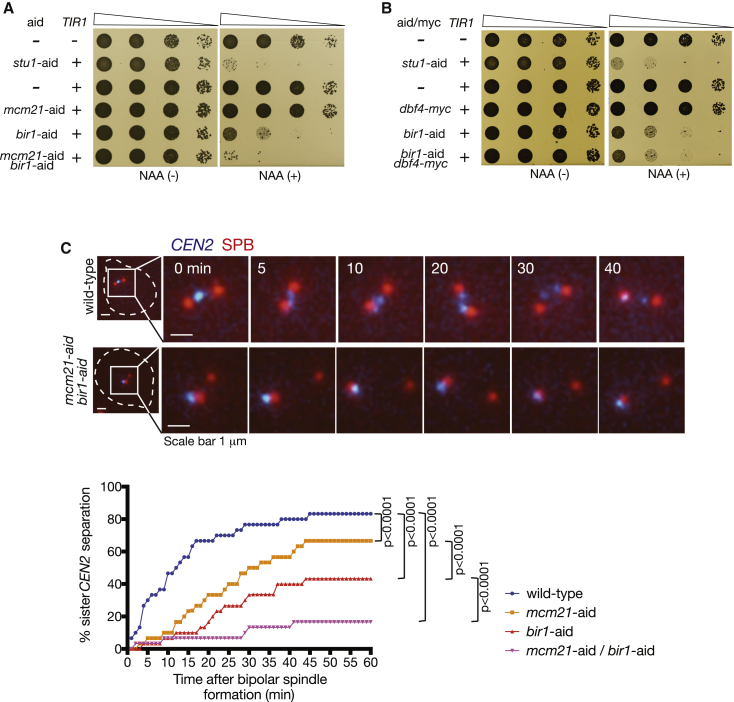
Bir1 and COMA Independently Promote Chromosome Bi-orientation (A) Bir1 depletion shows synthetic growth defects when combined with Mcm21 depletion. Yeast cells shown here were serially diluted (10 times each), spotted on plates, and incubated for 2 days in the presence (right) and absence (left) of 1-naphthaleneacetic acid (NAA). (B) Bir1 depletion shows no synthetic growth defects when combined with *dbf4-myc*. Yeast cells shown here were treated and analyzed as in (A). (C) Bir1 depletion and Mcm21 depletion cause further defects in chromosome bi-orientation when combined. *MCM21*^*+*^*BIR1*^*+*^ (wild-type; T12704), *mcm21-aid* (T12697), *bir1-aid* (T12698), and *mcm21-aid bir1-aid* (T12714) cells with *TIR*, *CEN2-tetOs*, *TetR-3*×*CFP*, and *SPC42-4*×*mCherry* were arrested in G1 with α-factor treatment and released into fresh medium. NAA was added 30 min before the release and also upon release. Microscopy images were acquired from 25 min after the release for 90 min at 1-min intervals. x axis shows time relative to separation of spindle pole bodies (SPBs) (Spc42-mCherry), which is defined as time 0. y axis shows % of cells showing separation of sister *CEN2s* on the bipolar spindle (i.e., after SPB separation) for at least two consecutive time points at or prior to indicated time points. n = 30 for each strain; p values were obtained using Kolmogorov-Smirnov test. See also Figure S1.

Because *bir1* mutations showed a stronger genetic interaction with *mcm21*Δ than with *ctf19Δ* at physiological culture conditions (20°C and 27°C) [11], we studied the Mcm21 function further. We fused *BIR1* and *MCM21* with the auxin-induced degron tag (*bir1-aid* and *mcm21-aid*) and investigated how this double depletion affected cell growth (Figures 1A and S1A). *STU1* is an essential gene, and *stu1-aid* was used as a control [14]. The *bir1-aid* suppressed cell growth in the presence of auxin, but not as completely as did *stu1-aid*. The *mcm21-aid* did not suppress growth on its own but, when combined with *bir1-aid*, showed further growth suppression than did *bir1-aid* alone (Figure 1A). A similar result was obtained using *bir1*Δ combined with *mcm21-aid* (Figure S1B). Thus, combined depletion (or deletion) of Bir1 and Mcm21 showed a synthetic growth defect.

The COMA complex promotes robust sister chromatid cohesion at peri-centromere regions by recruiting Dbf4-dependent kinase (DDK) to kinetochores [15, 16, 17, 18]. Robust peri-centromere cohesion is important for bi-orientation [15, 16, 19]. We addressed whether the above effects of Mcm21 depletion are due to a defect in peri-centromere cohesion. If so, a C-terminus-tagged *DBF4* (*dbf4-myc*), which impairs DDK recruitment to kinetochores [17], should show a similar defect to Mcm21 depletion. In fact, *dbf4-myc* produced a defect in peri-centromere cohesion in similar extent to (or slightly greater than) *mcm21* deletion (Figures S1C and S1D). However, in contrast to Mcm21 depletion, *dbf4-myc* showed no synthetic growth defect with Bir1 depletion (Figure 1B). This suggests that the synthetic growth defect of Mcm21-depletion and Bir1-depletion is not due to a defect in peri-centromere cohesion.

We analyzed the efficiency of chromosome bi-orientation establishment in wild-type control, individual Bir1 and Mcm21 depletions, and the double depletion. To assay this, we visualized a chosen centromere (*CEN2*) and spindle poles in live-cell fluorescence microscopy, scored the percentage of cells with bi-orientation (separation of sister *CEN2*), and plotted against time after formation of a bipolar spindle (Figure 1C). In a wild-type control, bi-orientation was established in the majority of cells within 15 min. Individual Mcm21 and Bir1 depletions showed moderate and substantial delays, respectively, in bi-orientation establishment. Intriguingly, Mcm21 and Bir1 double depletion showed a further delay in bi-orientation than did individual depletions. In the double depletion, only ∼17% of cells showed bi-orientation after 45 min. The effects of Mcm21 and Bir1 depletions seemed to be additive after 45 min, suggesting that COMA and Bir1 independently facilitate chromosome bi-orientation.

Note that, whereas essential COMA components Okp1-Ame1 facilitate outer kinetochore assembly, non-essential components Ctf19-Mcm21 have little such function [20, 21, 22]. It is therefore unlikely that the bi-orientation delay in Mcm21 depletion was due to reduced ability of the kinetochore for interacting with microtubules. Consistent with this, *CEN2* was always located in the vicinity of one spindle pole (i.e., interacted with microtubules) when bi-orientation was defective due to Mcm21 depletion or Mcm21 and Bir1 double depletion (Figure 1C, image, bottom).

### COMA Physically Interacts with Sli15 and Recruits Ipl1-Sli15 to the Inner Kinetochore, Independently of Bir1

**Figure 2:**
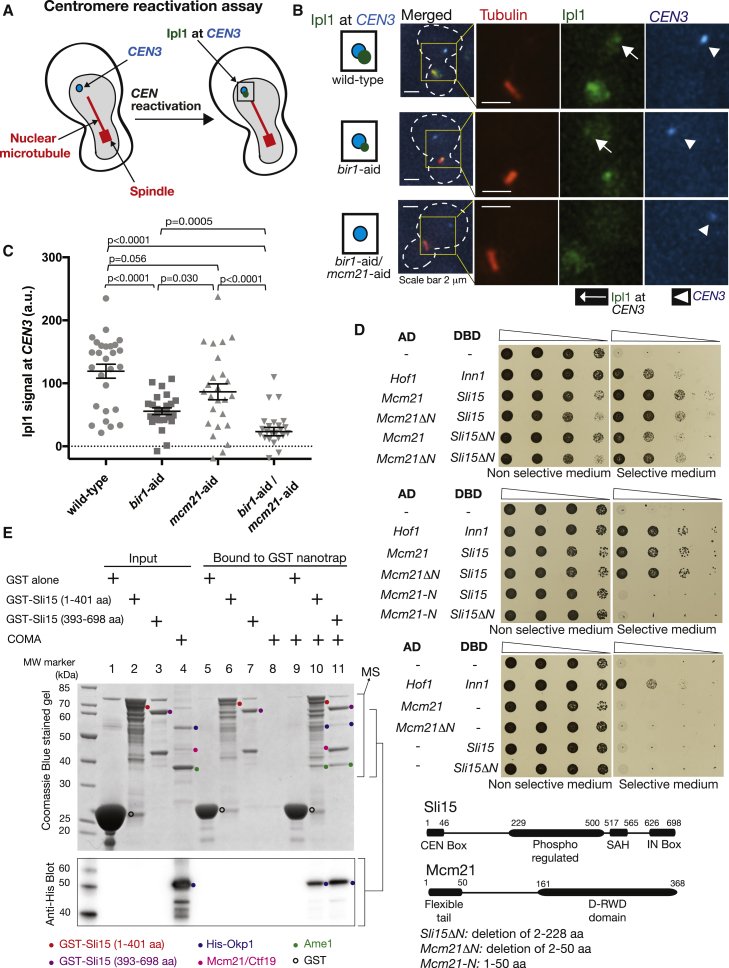
COMA Physically Interacts with Sli15 and Recruits Ipl1-Sli15 to the Inner Kinetochore Independently of Bir1 (A) Diagram shows the method of analyzing Ipl1 signals at isolated *CEN3*. *CEN3* under *GAL1-10* promoter was inactivated by transcription from the promoter, which prevented interaction with microtubules and placed it away from the spindle (left) [23, 24]. After reactivation of *CEN3* (by shutting off the promoter) during metaphase arrest, Ipl1 at *CEN3* was evaluated (right). See more details in STAR Methods. (B and C) Bir1 and COMA independently recruit Ipl1 to centromeres. *MCM21*^*+*^*BIR1*^*+*^ (wild-type; T12858), *mcm21-aid* (T12859), *bir1-aid* (T12860), and *mcm21-aid bir1-aid* (T12861) cells with *IPL1-GFP*, *TIR*, *GAL1-10* promoter*-CEN3-tetOs*, *TetR-3*×*CFP*, *mCherry-TUB1*, and *MET3* promoter-*CDC20* were treated and analyzed as in diagram in (A). Immediately after *CEN3* was reactivated, images were acquired for 10 min with a 1-min interval: (B) shows representative images of wild-type (top); Bir1-depleted (middle); and Bir1- and Mcm21-depleted (bottom) cells. Graph in (C) shows Ipl1 signals quantified at *CEN3* in n = 25–27 cells for each strain. Bars show means and SEMs. p values were obtained using t test. (D) Mcm21 and Sli15 interact in the yeast two-hybrid assay. The indicated constructs were fused to the Gal4 transcriptional activation domain (AD) and Gal4 DNA-binding domain (DBD). If the AD- and DBD-fused constructs physically interact, yeast cells grow on plates with selective medium. Yeast cells (10 times serial dilution) were incubated at 30°C for 2 days. Hof1 and Inn1 were used as a positive control [25]. Diagram shows domain structures of Sli15 and Mcm21 [26, 27]. (E) Recombinant COMA components are pulled down by immobilized recombinant Sli15. The following samples were run on the SDS-PAGE and stained with Coomassie blue (top): GST alone, indicated GST fusion proteins and COMA components (including His-tagged Okp1) were expressed in, and purified from, *E. coli* cells (lanes 1–4). GST and the GST fusion proteins were immobilized on GST Nanotrap; purified COMA components were added, washed, and proteins bound to GST Nanotrap were analyzed (lanes 9–11). Lanes 5–8 show controls. A bracket, marked “MS,” shows the area for mass spectrometry analyses (Figure S2C). A western blot (from a separate SDS-PAGE running the same samples) with anti-His antibody (to detect His1-Okp1) is shown below; connected brackets on the right show corresponding areas. Figure S2B shows the whole western blot. We interpret that colored dots indicate the proteins listed at bottom. The GST fusion proteins showed some truncation or degradation, and only their full-length bands are marked by colored dots. See also Figure S2.

Independent roles of COMA and Bir1 in facilitating chromosome bi-orientation may be due to their independent functions of recruiting Ipl1-Sli15 to centromeres or inner kinetochores. To address this possibility, we analyzed the localization of Ipl1 at centromeres. Because centromeres locate on the mitotic spindle, it was difficult to distinguish between Ipl1 localization on centromeres and on spindle microtubules. Therefore, to analyze Ipl1 localization specifically at a centromere, we isolated a chosen centromere (*CEN3*) from the spindle by inactivating it and thereby inhibiting kinetochore-microtubule interactions [23, 24] (Figure 2A, left). Subsequently, we reactivated *CEN3*, allowing its recapture by spindle microtubules (centromere reactivation assay). We analyzed Ipl1 localization at *CEN3* after reactivation but before recapture by spindle microtubules (Figures 2A, right, and 2B).

The level of Ipl1 signals at *CEN3* was quantified in wild-type control, individual Bir1 and Mcm21 depletions, and the double depletion (Figure 2C). The Ipl1 localization was marginally reduced by Mcm21 depletion and more clearly reduced by Bir1 depletion. Intriguingly, Mcm21 and Bir1 double depletion showed greater reduction of Ipl1 localization at *CEN3* compared with individual depletions. The effect of Mcm21 and Bir1 depletions on Ipl1 localization at *CEN3* seemed to be additive, suggesting that COMA and Bir1 independently promote Ipl1 localization at centromeres or kinetochores.

We also investigated the effect of *dbf4-myc*, which impairs peri-centromere cohesion [17], on Ipl1 localization on *CEN3* (Figure S2A). *DBF4* wild-type and *dbf4-myc* showed a similar level of Ipl1 localization at *CEN3*. Bir1 depletion reduced Ipl1 localization at *CEN3*, but this reduction was similar when combined with *DBF4* wild-type and *dbf4-myc*. These results with *dbf4-myc* contrast with those with Mcm21 depletion (Figure 2C), suggesting that reduced Ipl1 localization at centromeres (or kinetochores) with Mcm21 depletion in the absence of Bir1 was not due to weakened peri-centromere cohesion.

How then does COMA promote Ipl1 localization at centromeres or kinetochores, independently of Bir1? COMA may physically interact with Ipl1-Sli15 to enable their recruitment to the inner kinetochore, and we investigated possible physical interactions between Mcm21 and Sli15 using the yeast two-hybrid method (Figure 2D). Indeed, Mcm21 and Sli15 showed physical interaction, and this was not dependent on the Bir1-binding domain of Sli15 (Sli15 N terminus 1–228 amino acids [aa]) [28, 29] or the flexible Mcm21 N terminus (1–50 aa) [26].

We also addressed whether Sli15 directly interacts with COMA, using purified recombinant proteins. Glutathione S-transferase (GST)-Sli15 (1–401 aa), GST-Sli15 (393–698 aa), and COMA were separately expressed in bacteria and purified (Figure 2E, top, lanes 2–4). Both of the immobilized GST-Sli15 (1–401 aa) and GST-Sli15 (393–698 aa) pulled down COMA components, as analyzed by Coomassie blue staining (Figure 2E, top, lanes 5–11), western blots (Figures 2E, bottom, and S2B) and mass spectrometry (Figure S2C). Thus, COMA physically and directly interacts with Sli15, independently of Bir1, to recruit Ipl1-Sli15 to the inner kinetochore.

### Engineered Recruitment of Ipl1-Sli15 to the Inner Kinetochore Restores Bi-orientation when Both COMA and Bir1 Are Defective

Our results suggest that the level of Ipl1 localization at centromeres is correlated well with efficiency of chromosome bi-orientation when Bir1 and Mcm21 were depleted individually and in combination (Figures 1C and 2C). In particular, with Bir1 and Mcm21 double depletion, both bi-orientation and Ipl1 localization at centromeres (or inner kinetochores) were almost completely abolished. This raises the possibility that Ipl1-Sli15 localization at centromeres or inner kinetochores is crucial for chromosome bi-orientation. One way to test this is to engineer recruitment of Ipl1-Sli15 to centromeres or inner kinetochores in the absence of Bir1 and Mcm21 and to test whether this can rescue bi-orientation.

**Figure 3:**
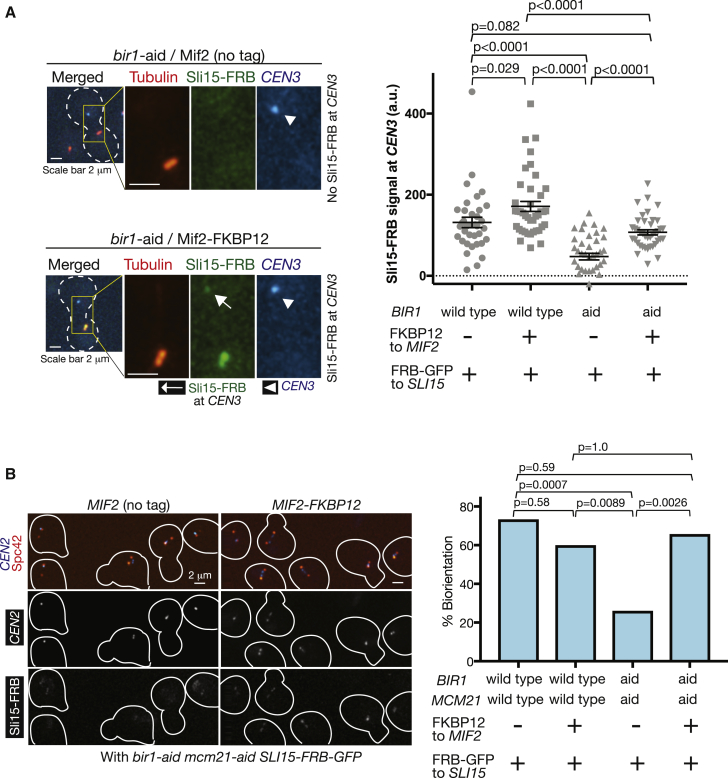
Engineered Recruitment of Sli15 to the Inner Kinetochore Restores Bi-orientation when Both COMA and Bir1 Are Defective (A) FKBP12-fused Mif2 recruits FRB-fused Sli15 to isolated *CEN3*. (a) *BIR1+* (wild-type) or *bir1-aid* and (b) *MIF2* with or without fusion to *FKBP12* were combined as indicated below the graph (T13199–T13202 from left to right). All strains carried *SLI15-FRB-GFP*, *TIR*, *TOR1-1, fpr1Δ, GAL1-10* promoter*-CEN3-tetOs*, *TetR-3*×*CFP*, *mCherry-TUB1*, and *MET3* promoter-*CDC20.* Cells were treated as in Figure 2B, except that rapamycin was added 30 min before the start of image acquisition. Microscope images (left) show representative examples of *bir1-aid MIF2* (no tag; top) and *bir1-aid MIF2-FKBP12* (bottom) cells. Sli15-FRB signals were quantified at *CEN3* in n = 26–30 cells for each strain (graph at right). Bars show means and SEMs. p values were obtained by t test. (B) Engineered Sli15 association with Mif2 restores bi-orientation when both COMA and Bir1 are defective. (a) *BIR1*^*+*^*MCM21*^*+*^ (wild-type) or *bir1-aid mcm21-aid* and (b) *MIF2* with or without fusion to *FKBP12* were combined as indicated below the graph (T13438, T13441, T13440, and T13444 from left to right). All strains carried *SLI15-FRB-GFP*, *TIR*, *TOR1-1, fpr1Δ, CEN2-tetOs*, *TetR-3*×*CFP*, *SPC42-4*×*mCherry*, and *MET3* promoter-*CDC20.* They were cultured in methionine drop-out medium, arrested in G1 with α-factor treatment, and released into YPAD plus 2 mM methionine, leading to metaphase arrest (due to Cdc20 depletion). At 2 h following the release, microscopy images were acquired. Rapamycin was added 30 min before the start of image acquisition. Representative images are shown on left; cell shapes are shown in white lines. y axis of the graph (right) shows % of sister *CEN2* separation, representing its bi-orientation. n = 30–35 for each strain; p values were obtained using Fisher’s exact test. See also Figures S3A and S3B.

To engineer Ipl1-Sli15 recruitment to inner kinetochores, we used the rapamycin-dependent association between FRB and FKBP12 [30] and fused FRB and FKBP12 to Sli15 and Mif2, respectively. Mif2 is the yeast CENP-C ortholog and an inner kinetochore component and was chosen for this purpose because its inner kinetochore localization would not be affected by Mcm21 depletion [22]. As in Figure 2A, we isolated *CEN3* from the mitotic spindle and studied Sli15-FRB localization at *CEN3* in the presence of rapamycin (Figure 3A). When Mif2 was not fused to FKBP12 (control), Bir1 depletion considerably reduced the level of Sli15-FRB at *CEN3*, consistently with Figure 2B. Fusion of FKBP12 to Mif2 rescued Sli15-FRB localization at *CEN3* to an almost normal level, when Bir1 was depleted (Figure 3A). Thus, indeed, Sli15 is recruited to the inner kinetochore by this engineered system.

We next evaluated efficiency of chromosome bi-orientation with the engineered recruitment of Sli15 to inner kinetochores. As in Figure 3A, FRB and FKBP12 were fused to Sli15 and Mif2, respectively, and bi-orientation frequency was evaluated by visualizing *CEN2* on the spindle in the presence of rapamycin. When Mif2 was not fused to FKBP12 (control), very little Sli15-FRB co-localized with *CEN2* (Figures 3B, image, left, and S3A), and Bir1 and Mcm21 double depletion significantly reduced frequency of bi-orientation (Figure 3B, graph; as also shown in Figure 1C). Importantly, in the Bir1 and Mcm21 double depletion, fusion of FKBP12 to Mif2 enhanced Sli15-FRB co-localization with *CEN2* (Figures 3B, image, right, and S3A) and rescued frequency of bi-orientation to an almost normal level (Figure 3B, graph). Thus, engineered recruitment of Sli15 to inner kinetochores rescued bi-orientation when both Bir1 and COMA were defective. Intriguingly, the engineered Sli15 recruitment made cells inviable even with wild-type Bir1 and Mcm21, which we speculate is due to abnormally sustained localization of Ipl1-Sli15 at inner kinetochores during anaphase (Figure S3B). Overall, the above results suggest that Ipl1-Sli15 localization at centromeres or inner kinetochores is crucial for chromosome bi-orientation.

### Ipl1 Still Localizes at the Inner Kinetochore with *bir1*Δ *sli15*ΔN, which Is Dependent on COMA

**Figure 4:**
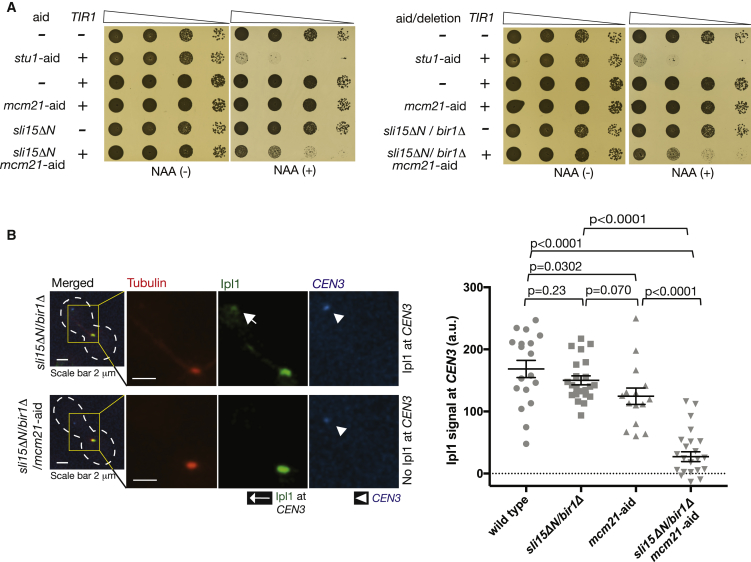
Ipl1 Still Localizes at the Inner Kinetochore with *bir1*Δ *sli15*ΔN, and This Is Dependent on COMA (A) *bir1*Δ *sli15*ΔN (*sli15*ΔN; deletion of 2–228 aa) shows synthetic growth defects when combined with Mcm21 depletion. Yeast cells shown here were serially diluted (10 times each), spotted on plates, and incubated for 2 days in the presence (right) and absence (left) of NAA. (B) Ipl1 localizes at the inner kinetochore with *bir1*Δ *sli15*ΔN dependent on COMA. *BIR1*^*+*^*SLI15*^*+*^*MCM21*^*+*^ (wild-type; T12248), *bir1*Δ *sli15*Δ*N* (T12229), *mcm21-aid TIR* (T12738), and *bir1*Δ *sli15*Δ*N mcm21-aid TIR* (T12739) cells with *IPL1-GFP*, *GAL1-10* promoter*-CEN3-tetOs*, *TetR-3*×*CFP*, *mCherry-TUB1*, and *MET3* promoter-*CDC20* were treated and analyzed as in Figure 2B. Immediately after reactivation of *CEN3*, images were acquired for 10 min with a 1-min interval. Representative images (left) of T12229 and T12739 cells are shown at top and bottom, respectively. Ipl1 signals were quantified at *CEN3* in n = 15–25 cells for each strain (graph at right). Bars show means and SEMs. p values were obtained using t test. See also Figure S3C.

It was previously reported that, when Sli15 lacks its Bir1-binding domain 2–228 aa (*sli15*ΔN), yeast cells still establish bi-orientation and grow almost normally in the absence of Bir1 [10]. Based on this, it was proposed that Ipl1-Sli15 localization at centromeres is dispensable for chromosome bi-orientation [10]. Given our finding that COMA promotes recruitment of Ipl1-Sli15 to inner kinetochores independently of Bir1 (Figure 2B), bi-orientation and cell growth in *bir1*Δ *sli15*ΔN may be dependent on COMA. Consistent with this, cell growth of *bir1*Δ *sli15*ΔN cells was severely reduced when combined with Mcm21 depletion (Figure 4A). In contrast, growth of *bir1*Δ *sli15*ΔN cells was not affected when combined with *dbf4-myc* (Figure S3C), which showed a defect in peri-centromere cohesion (Figure S1D). Therefore, the effect of Mcm21 depletion on the growth of *bir1*Δ *sli15*ΔN-cells was not due to weakened peri-centromere cohesion.

We next examined localization of Ipl1 on *CEN3*, which was isolated from the spindle as in Figure 2A. Intriguingly, with *bir1*Δ *sli15*ΔN, Ipl1 localization at *CEN3* was similar to that with the wild-type control (Figure 4B). This result contrasts with Ipl1 localization at *CEN3* with Bir1 depletion alone, cells of which showed considerable reduction of Ipl1 there (Figure 2B). This suggests that *sli15*ΔN enhances Ipl1 localization at centromeres in the absence of Bir1, though the mechanism for this is still unclear. In any case, when combined with Mcm21 depletion, the *bir1*Δ *sli15*ΔN cells showed a very severe defect in Ipl1 localization at *CEN3* (Figure 4B). In conclusion, Ipl1 still localizes at the inner kinetochore with *bir1*Δ *sli15*ΔN, and this localization depends on COMA.

### Implications of This Study

In this study, we have demonstrated that the COMA kinetochore sub-complex physically interacts with Sli15 and recruits Ipl1-Sli15 to the inner kinetochore, independently of Bir1. Physical interactions between Sli15 and COMA components (Ctf19 and Mcm21) have also been shown in a recently posted preprint, using chemical crosslinking of recombinant proteins [31]. Furthermore, using an engineered system for recruiting Ipl1-Sli15 to the inner kinetochore when both Bir1 and COMA are defective, we were able to show that localization of Ipl1-Sli15 at centromeres or inner kinetochores is crucial for bi-orientation in budding yeast. We presume that the centromere and inner kinetochore are suitable locations of Ipl1-Sli15 to enable efficient phosphorylation of outer kinetochores that drives error correction.

Our finding also gives insight into how tension across sister kinetochores halts the Aurora B action of disrupting kinetochore-microtubule interaction. One popular model for this is the spatial separation model, i.e., tension causes kinetochore stretching, which moves outer kinetochore substrates away from Aurora-B-localizing sites at centromeres [3, 5, 9]. Consistent with this model, Aurora B delocalizes from outer kinetochores when bi-orientation is established in budding yeast [3, 32], outer kinetochores (but not centromeres) are under differential regulation by Aurora B activity before and after establishment of bi-orientation in human cells [33], and outer kinetochore components are dephosphorylated when tension is applied [34, 35]. The spatial separation model predicts that Aurora B localization at centromeres is required for bi-orientation. However, it has been shown that bi-orientation can be established in the absence of Bir1 in budding yeast [10], and this has raised the possibility that Ipl1 (Aurora B) localization at centromeres is dispensable for bi-orientation—if so, that might rule out the spatial separation model. Our results suggest that the COMA-dependent mechanism still recruits Ipl1 (Aurora B) to the inner kinetochore in the absence of Bir1, at least partially compensating for its centromere localization and supporting bi-orientation. If our conclusion is correct, the spatial separation model still remains a plausible model for Aurora-B-driven error correction, at least, in budding yeast.

On the other hand, our data do not exclude other models explaining how tension halts the Aurora B action of disrupting kinetochore-microtubule interaction. For example, the kinetochore-microtubule interface may form a stable structure by itself when tension is applied [36], which may overcome the Aurora B action. Localization of Aurora B at the outer kinetochore may have crucial roles in rendering its action tension dependent [37, 38]. Aurora B kinase activity or counteracting phosphatase activity may be directly regulated by tension [39, 40]. Our finding that Ipl1 (Aurora B) localization at the centromere or inner kinetochore is crucial for bi-orientation may also shed new light on these models.

In this study, we used budding yeast as a model organism. Are the conclusions in this study also the case in vertebrate cells? Ipl1, Sli15, Bir1, and COMA are conserved from yeast to vertebrates, and their vertebrate counterparts are called Aurora B, INCENP, survivin, and CENP-O/P/Q/U, respectively [41, 42]. The complex consisting of Aurora B, INCENP, survivin, and Borealin (Nbl1 in yeast) is called the chromosomal passenger complex [43]. Crucially, it was recently reported that INCENP lacking its survivin-binding domain (N terminus) still supports bi-orientation in human cells [44], as does yeast Sli15 lacking its N terminus [10]. If mechanisms were conserved between yeast and vertebrate cells, CENP-O/P/Q/R would promote recruitment of Aurora B-INCENP to inner kinetochores independently of survivin. Intriguingly, in the *Xenopus* egg extract system, the inner kinetochore was not fully assembled when INCENP lacked its N terminus (presumably, in contrast to human cells); in this circumstance, this INCENP mutant could no longer support bi-orientation, even if the outer kinetochore assembly seemed normal [45]. Therefore, inner kinetochore components may indeed support Aurora B-INCENP localization and error correction, independently of survivin, in vertebrate cells.

## STAR★Methods

### Key Resources Table

**Table:** 

REAGENT or RESOURCE	SOURCE	IDENTIFIER
**Antibodies**		

Anti-AID antibody, sheep polyclonal	Karim Labib lab	N/A
Anti-6His tag antibody, mouse monoclonal (HIS.H8)	abcam	ab18184
Anti-Sheep IgG (whole molecule)–Peroxidase antibody produced in donkey	Sigma Aldrich	A3415
Goat anti-mouse IgG H&L, HRP	abcam	ab97023

**Bacterial and Virus Strains**		

One Shot TOP10 Chemically Competent Cells	Invitrogen	C404003

**Chemicals, Peptides, and Recombinant Proteins**		

1- Naphthaleneacetic acid (NAA)	Sigma-Aldrich	N0640-25G
α-factor	Pepceuticals Ltd	N/A
Rapamycin	LC Laboratories	R-5000
GST-Trap Agarose	ChromoTek	sta-10
HisPur Nic-NTA Superflow agarose	ThermoScientific	25214
Glutathione Sepharose 4 Fast Flow	Sigma	GE17-5132-01
Concanavalin A	Sigma-Aldrich	C7275

**Experimental Models: Organisms/Strains**		

*S. cerevisiae* W303 and its derivatives (see Table S1 for detail)	This study	See Table S1
PJ69-4A	Philip James	See [46]

**Recombinant DNA**		

pGADT7 (*LEU2* marker)	Clontech Takara	630442
pGBKT7 (*TRP1* marker)	Clontech Takara	630443
pGADT7-*HOF1* (*LEU2* marker)	Karim Labib lab	pKL772
pGBKT7-*INN1* (*TRP1* marker)	Karim Labib lab	pKL769
pGADT7-*MCM21* (*LEU2* marker)	This study	pT3294
pGADT7-*MCM21ΔN* (*LEU2* marker)	This study	pT3295
pGADT7-*MCM21−N* (*LEU2* marker)	This study	pT3296
pGBKT7-*SLI15* (*TRP1* marker)	This study	pT3297
pGBKT7-*SLI15ΔN* (*TRP1* marker)	This study	pT3298
pRS405-*SLI15ΔN* (*URA3* marker)	This study	pT3299
pGEX6P-1-*GST-SLI15* (1-401)	This study	pT3176
pGEX6P-1-*GST-SLI15* (393-698)	This study	pT3177
pETDuet-*His-OKP1-AME1*	This study	pT3299
pRSFDuet-*CTF19-MCM21*	This study	pT3333

**Software and Algorithms**		

Volocity 6.1.1	PerkinElmer	http://www.perkinelmer.com/
Prism 6.0	Graph-Pad	https://www.graphpad.com/
ApE A plasmid editor	M. Wayne Davis	http://jorgensen.biology.utah.edu/wayned/ape/
SoftWoRx 6.5.2	GE Healthcare	http://incelldownload.gehealthcare.com/bin/download_data/SoftWoRx/6.5.2/SoftWoRx.htm
Mascot 2.6	Matrix Science	http://www.matrixscience.com
ImageLab 4.1	Bio-Rad	http://www.bio-rad.com/en-uk/product/image-lab-software?ID=KRE6P5E8Z

**Other**		

Glass-bottom dish	MatTek	P35G-1.5-10-C P35G-1.5-14-C

### Contact for Reagent and Resource Sharing

Further information and requests for resources and reagents should be directed to and will be fulfilled by the Lead Contact, Tomoyuki Tanaka (t.tanaka@dundee.ac.uk).

### Experimental Model and Subject Details

#### Yeast strains and cell culture

The background of yeast strains (W303) and the methods for yeast culture have been described previously [47, 48]. To synchronize cells in the cell cycle, yeast cells were arrested in G1 phase by treatment with yeast mating pheromone (α-factor) and subsequently released to fresh media [47]. Cells were cultured at 25°C in YPA medium containing 2% glucose (YPAD) unless otherwise stated. Constructs of *GAL1-10* promoter*-CEN3-tetOs* [23, 49, 50], *TetR-3*×*CFP* [49, 51], *MET3* promoter-*CDC20* [52], *mCherry-TUB1* [53], *SPC42-4*×*mCherry*, *NIC96-4*×*mCherry* [54], *CEN2*-*tetO*s (*tetO*×224 was inserted 600 bp away from *CEN2*) [17] and *tetO*s at 15 kb from *CEN12* [17] were described previously. To generate *bir1*Δ and *mcm21*Δ, the whole coding region of the gene was replaced with the *Kl. LEU2* and *KAN-MX4* cassette, respectively, using a one-step PCR procedure [55], i.e., the selection marker was amplified by PCR primers carrying additional 50-mer single-strand DNAs that are homologous to 5′ and 3′ regions of the gene, and introduced into yeast cells by transfection, followed by selection of cells with targeted gene deletion. *IPL1* was tagged with yEGFP at its C terminus at its original locus using the yEGFP-SpHIS5 cassette (pKT128) as a PCR template using a one-step PCR procedure [56], i.e., *yEGFP* and the selection marker were amplified by PCR primers carrying additional 50-mer single-strand DNAs that are homologous to the end of the coding region and 3′ region of the gene, and introduced into yeast cells by transfection, followed by selection of cells with the *IPL1-yEGFP* fusion gene. *sli15*ΔN (deletion of 2-228 aa) replaced the original *SLI15+* wild-type gene at its original locus, using the loop-in and loop-out strategy, as follows; 1) *SLI15* promoter, the ATG start codon and *SLI15* coding sequence corresponding to 229–465 aa were cloned into pRS405 vector (URA3 marker) (pT3299), 2) the construct was integrated (loop-in) at *SLI15* locus by homologous recombination after cutting at the *NruI* site within the *SLI15* promoter, 3) the strain was grown with 5-FOA to remove the *URA3* marker (loop-out) by homologous recombination, and 4) strains were checked by PCR and DNA sequencing to select those with *sli15*ΔN (deletion of 2-228 aa) and without the original *SLI15*^+^ wild-type gene. The genotype of strains used in this study is provided in Table S1. The strain numbers that are not included in Figure legends are as follows: Figure 1A (T7107, T10133, T9030, T12410, T12401 and T12614 from top to bottom), Figure 1B (T7107, T10133, T9030, T12809, T12401 and T12810 from top to bottom), Figure 4A left (T7107, T10133, T9030, T12410, T12064 and T12467 from top to bottom), Figure 4A right (T7107, T10133, T9030, T12410, T12066 and T12466 from top to bottom), Figure S1B (T7107, T10133, T9030, T12410, T12099 and T12613 from top to bottom) and Figure S3C (T9030, T12809, T12066 and T12811 from top to bottom).

#### Depletion of AID-tagged proteins

To deplete Bir1 and Mcm21, *BIR1 and MCM21* were fused to an *aid* tag (auxin-inducible degron tag) at their C-termini at the original loci in the strain carrying the rice F-box gene *TIR1* [57]. In the presence of auxin NAA (1-Naphthaleneacetic acid; 2 mM on plates and 0.5 mM in liquid media, unless otherwise stated), *aid*-tagged proteins interact with the SCF E3 ubiquitin ligase, mediated by Tir1, which leads to their ubiquitylation and degradation by the proteasome [57]. To detect Bir1-aid and Mcm21-aid in western blots, an anti-AID tag sheep antibody (generated by MRC PPU Reagents and Services, University of Dundee, and was kindly provided by Karim Labib) was used.

#### Engineered association between proteins

To engineer association between Sli15 and Mif2, *SLI15* was tagged with *FRB-GFP* using a *FRB-GFP-kanMX6* (pFA6a-FRB-GFP-kanMX6) cassette, and *MIF2* with *2×FKBP12-TRP1* cassette (pFA6a-2×FKBP12-TRP1) at their C-termini at their original gene loci by a one-step PCR method [58]. For this experiment, yeast strains also carried *TOR1-1*, which conferred rapamycin resistance, and *fpr1*Δ mutations. Association of *FRB* and *FKBP12* fusion proteins was induced by addition of 1 μM of rapamycin to culture media.

### Method Details

#### Microscopy image acquisition

During time-lapse imaging, yeast cells were immobilized on a glass-bottomed dish (MatTek, P35G-1.5-10-C) coated with concanavalin A (Sigma C7275), and maintained in synthetic-complete (SC) plus YPA medium (3:1 ratio) [24, 47]. For imaging during metaphase arrest of cells with *MET3* promoter-*CDC20, 2* mM methionine was added to the medium to ensure Cdc20 depletion. Where relevant, NAA was added to the medium during imaging to maintain protein degradation, and Rapamycin was added to the medium during imaging to maintain FRB–FKBP12 interaction. Images were acquired using a DeltaVision Elite microscope (Applied Precision), an UPlanSApo 100 × objective lens (Olympus; NA 1.40), SoftWoRx software (Applied Precision), and a CoolSnap HQ (Photometrics). We acquired 7–11 (0.7 μm apart) z sections, which were subsequently processed through deconvolution, and analyzed with Volocity (Improvision) software. CFP, GFP, and mCherry signals were discriminated using the 89006 multi-band filter set (Chroma). For the image panels in Figures, Z sections were projected to two-dimensional images.

#### Centromere reactivation assay

To analyze Ipl1 or Sli15 localization at a centromere isolated from the spindle, the centromere re-activation assay was used [23, 24]. In this assay, kinetochore assembly was delayed on a chosen centromere by transcription from the *GAL* promoter (*GAL1-10* promoter*-CEN3-tetOs* replacing *CEN15* on chromosome *XV*). This increased the distance between the centromere and the mitotic spindle, allowing observation of protein localization specifically at *CEN3* after inducing kinetochore assembly on the centromere by turning off the *GAL* promoter in metaphase-arrested cells. Cells with *GAL1-10* promoter*-CEN3-tetOs* and *MET3* promoter-*CDC20* were cultured overnight in methionine drop-out media with 2% raffinose, treated with α-factor for 2.5 hours (to arrest in G1 phase), and released to fresh media with 2% raffinose, 2% galactose and 2 mM methionine (for Cdc20 depletion and *CEN3* inactivation). After 2 hours, cells were suspended in SC medium containing 2% glucose and methionine to reactivate *CEN3*. Protein localization was analyzed at *CEN3* after *CEN3* reactivation and before *CEN3* interaction with microtubules extended from the spindle. After Z sections were projected to 2D images, Ipl1-GFP and Sli15-FRB-GFP signals (colocalizing at *CEN3*) were quantified in the area of 2x2 pixels at their maximum intensity, using the Voxel Spy tools of Volocity. Background was subtracted in every measurement.

#### Yeast two-hybrid assay

Two-Hybrid analysis was carried out as in [25]. Briefly, the assay was based on the Gal4 transcription factor and performed after co-transformation of derivatives of pGADT7 (Gal4 activation domain; *LEU2* marker; Clontech) and pGBKT7 (Gal4 DNA binding domain; *TRP1* marker; Clontech) into the yeast strain PJ69-4A (two-hybrid strain [46]). For each assay, independent colonies from the transformation were mixed together in water and spotted in ten-fold dilutions onto SC medium lacking tryptophan and leucine (selective for pGADT7 and pGBKT7, but non-selective for the two-hybrid interaction) and SC medium lacking tryptophan, leucine, histidine and adenine (selective for the two-hybrid interaction).

#### Purification of GST-Sli15 (1-401 aa) and GST-Sli15 (393-698 aa)

*SLI15* coding DNAs, corresponding to 1–401 aa and 393-698 aa, were fused with GST on pGEX6P-1 (GE Healthcare), which were named pT3176 and pT3177, respectively. The pT3176 and pT3177 were introduced into RosettaGami2 *E. coli* cells (Novagen). The *E. coli* cells were grown in LB medium, and the GST fusion protein was expressed at 18°C with 0.1 mM IPTG induction overnight. Cells were harvested and disrupted using an Emulsiflex cell disruptor (ATA Scientific) in the lysis buffer (50 mM HEPES-NaOH pH7.6, 1 M NaCl, 5 mM β-mercaptoethanol, 1% Triton X-100) supplemented with cOmplete protease inhibitors (Roche). The cleared lysate was loaded onto 1ml GSTrap FF column (GE Healthcare). Subsequently the proteins, trapped on the column, were eluted with 40 mM reduced glutathione (Sigma) in the elution buffer (50 mM HEPES-NaOH pH7.6, 0.3 M NaCl, 1 mM DTT, 0.05% NP-40, 10% glycerol). The eluate was loaded onto Superdex 200 10/300 column (GE Healthcare) equilibrated with 50 mM HEPES-NaOH pH7.6, 0.3 M NaCl, 1 mM DTT, 0.05% NP-40, 15% glycerol, and fractions containing full-length GST-Sli15 (1–401 aa) and GST-Sli15 (393-698 aa) were pooled and concentrated.

#### Purification of recombinant COMA

DNA fragments coding for *His-OKP1* and *AME1* were cloned into the pETDuet (Amp) vector (Novagen), while coding regions for *CTF19* and *MCM21* were cloned into the pRSFDuet (Kan) vector (Novagen). The two constructs (pT3329 and pT3333, respectively) were introduced together into RosettaGami2(DE3)pLysS *E. coli* cells (Novagen), and protein expression was induced with 0.2 mM IPTG at 16°C overnight. Cells were lysed in buffer 50 mM Tris-HCl pH 7.5, 250 mM NaCl, 0.5% Igepal CA-630, 10 mM β-glycerophosphate, 5% glycerol, 10 mM imidazole and 1 mM PMSF, supplemented with cOmplete protease inhibitors (Roche). The cleared lysate was incubated with His-Pur Nic-NTA Superflow agarose (Thermo) for 1.5 h at 4°C. Subsequently, the agarose beads were washed with 5CV wash buffer (50 mM Tris-HCl pH 7.5, 250 mM NaCl, 0.5% Igepal CA-630 and 20 mM imidazole). The proteins bound on the agarose were eluted in buffer containing 50 mM Tris-HCl pH 7.5, 100 mM NaCl, 0.1% Triton X-100, 1 mM β-mercaptoethanol, 250 mM imidazole and 1 mM PMSF, supplemented with cOmplete protease inhibitors (Roche). Elution fractions were pooled and further purified by gel filtration chromatography (HiLoad 16/60 Superdex 200, GE Healthcare). The gel filtration buffer contained 20 mM HEPES-NaOH pH7.5, 300 mM NaCl, 10% glycerol, 1mM DTT and 1 mM PMSF. The presence of His-Okp1, Ame1, Ctf19 and Mcm21 in the elution peak was confirmed by mass spectrometry (MS/MS) after trypsin digestion, and by western blot with anti-His antibody (Abcam).

#### GST pull-down assay

GST alone, GST-Sli15 (1-401) or GST-Sli15 (393-698) were incubated together with purified COMA in buffer containing 50 mM HEPES-NaOH pH 7.4, 150 mM NaCl, 5% glycerol, 0.05% Igepal, 2 mM DTT and 0.5 mM EDTA for 20 min at 4°C. This was added to GST-Trap Agarose (sta-10, ChromoTek) and pre-equilibrated with the above buffer. After 1-hour incubation at 4°C with rotation, the beads were washed three times with the above buffer. For elution of the bound proteins, beads were boiled in 2x SDS-PAGE sample buffer. Eluted proteins were separated by SDS–PAGE (Novex 10% Bis-Tris gel), and stained with Coomassie Blue (Imperial Blue, ThermoScientific). We confirmed that the band with the expected size of His-Okp1, visible on the Coomassie-stained gel (Figure 2E, lane 4, 10 and 11), indeed contained His-Okp1 using western blot with anti-His antibody (Abcam). Two SDS-PAGEs ran the same samples (in the same amount) in parallel; one was used for the western blot as mentioned above while the other was used for mass spectrometry analyses (see next section).

#### Mass spectrometry analysis of pull-down samples

After Coomassie staining, gel pieces were excised from lanes 2, 3, 4, 9, 10 and 11, comprising the expected protein sizes (33 - 75kDa) to cover GST-Sli15 (1-401) (72 kDa), GST-Sli15 (393-698) (60 kDa), His-Okp1 (49 kDa), Mcm21 (43 kDa), Ctf19 (43 kDa) and Ame1 (37 kDa). After destaining the gel pieces, proteins were reduced with 10 mM DTT for 45min at 55°C and alkylated with 55 mM iodoacetamide for 30min at ambient temperature. Digestion was performed with mass-spectrometry grade trypsin (Pierce) at 37°C, once for 4 h, then overnight. Subsequently, peptides were extracted by two rounds of sonication in 50%ACN in 0.1%TFA and one round of sonication in 70%ACN in 0.1%TFA. Pooled extracts of each sample were dried completely, re-suspended in the appropriate solvent and subjected to mass spectrometry. Measurement was performed using a Q Exactive HF, and data were analyzed with Mascot 2.4.0.

### Quantification and Statistical Analysis

All the experiments were repeated at least twice and similar results were obtained. Statistical analyses were carried out using Prism software (Graphpad). Methods of statistic tests are stated in each relevant figure legend. Kolmogorov–Smirnov test was used in Figure 1C to test if efficiency in bi-orientation establishment is different between two groups. t test was used in Figures 2C, 3A, 4B, S2A, and S3A to test if there is a significant difference between the means of two groups. Fisher’s exact test was used in Figures 3B and S1D to test if frequency of bi-orientation (Figure 3B) and sister separation (Figure S1D) is significantly different between two groups. The null hypotheses in these tests were that the samples were collected randomly and independently from the same population. All *p* values were two-tailed, and the null hypotheses were reasonably discarded when *p* values were < 0.05. Sample numbers are also stated in figure legends.

## Supplemental Information

Document S1. Figures S1–S3 and Table S1

Document S2. Article plus Supplemental Information
