# Supplementary material for: Aurora B-INCENP Localization at Centromeres/Inner Kinetochores Is Required for Chromosome Bi-orientation in Budding Yeast
Source: Curr Biol. 2019 May 6;29(9):1536–1544.e4. doi: 10.1016/j.cub.2019.03.051 (PMC6509284; doi:10.1016/j.cub.2019.03.051)
Supplement: Document S1. Figures S1–S3 and Table S1 [file mmc1.pdf]

Current Biology, Volume *29*

Supplemental Information

Aurora B-INCENP Localization at Centromeres/Inner Kinetochores Is Required for Chromosome Bi-orientation in Budding Yeast

Luis J. García-Rodríguez, Taciana Kasciukovic, Viola Denninger, and Tomoyuki U. Tanaka

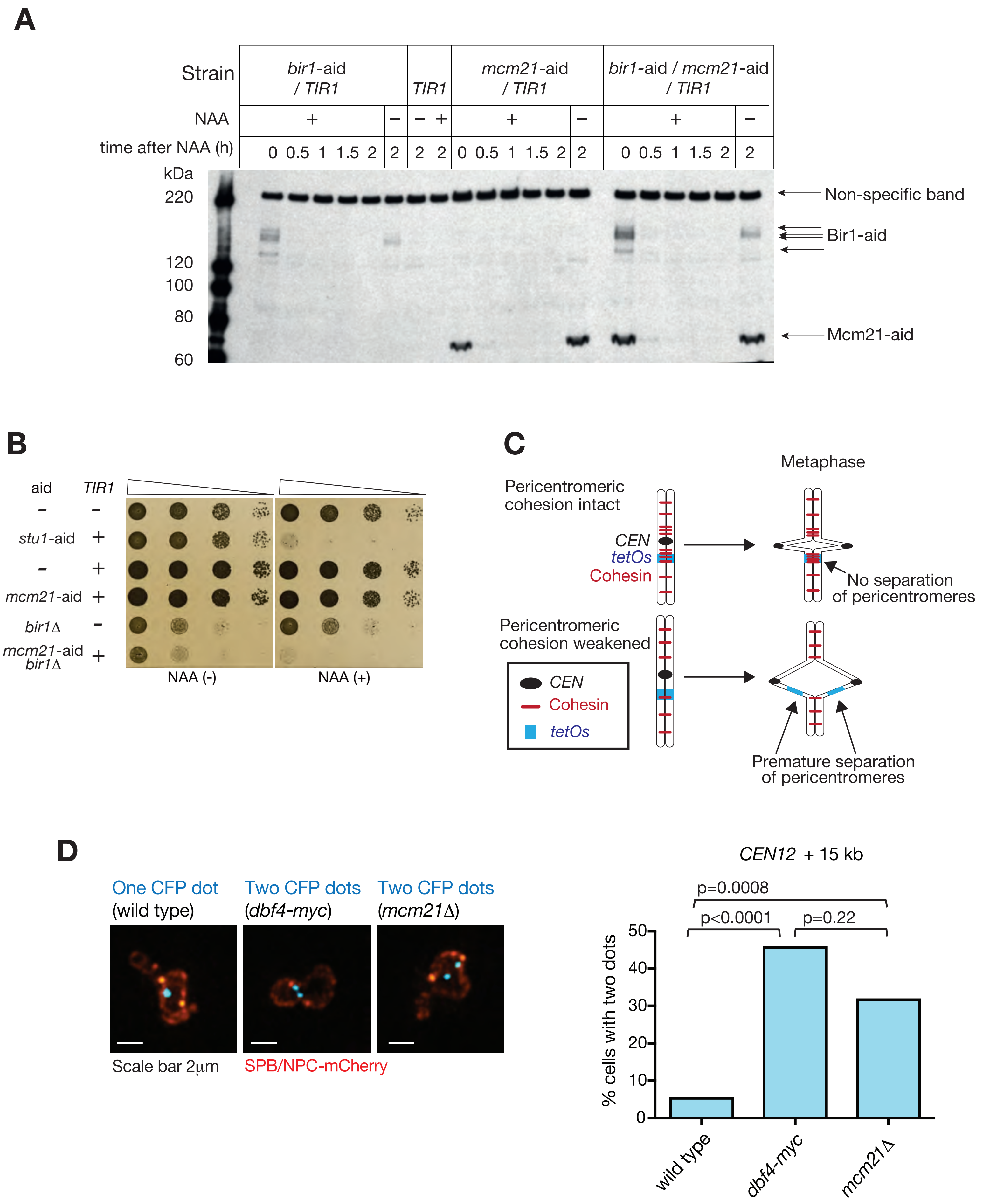

(Figure S1, legend on next page)

Figure S1. Evaluation of protein depletion, synthetic growth defects and peri-centromere cohesion. Related to Figure 1.

A. Bir1-aid and Mcm21-aid are depleted to undetectable levels 30 minutes after addition of NAA. Yeast cells carrying *TIR* with *mcm21-aid* and *bir1-aid* individually and in combination were collected every 0.5 h after addition of 2 mM NAA (or 2 h without NAA, as a control) as indicated. Proteins were separated by the SDS-PAGE (NuPAGE 3-8 % Tris-Acetate gel), blotted and probed with an anti-AID tag antibody. The bands corresponding to Bir1-aid and Mcm21-aid are indicated on the right. The non-specific band of ~220 kDa served as a loading control.

B. Bir1 deletion shows synthetic growth defects when combined with Mcm21 depletion. Yeast cells with *mcm21-aid TIR* and *bir1*Δ individually and in combination, were serially diluted (10 times dilution each), spotted on plates and incubated for 2 days in the presence (right) and absence (left) of NAA. Cells without AID tag or with *stu1-aid* were analysed in the same way as controls.

C. Diagram illustrates that sister *tetO*s at peri-centromeres (e.g. at 15 kb from *CEN12*, see **D**) shows frequent separation during metaphase when peri-centromere cohesion is weakened.

D. *dbf4-myc* shows a defect in peri-centromere cohesion to a similar extent to (or marginally greater than) *mcm21* deletion. *DBF4*$^{+}$ *MCM21*$^{+}$ (wild-type, T10141), *dbf4-myc* (T10142) and *mcm21*Δ (T13365) cells with *tetO*s at 15kb from *CEN12*, *TetR-3×CFP*, *MET3* promoter-*CDC20*, *SPC42-4×mCherry* and *NIC96-4×mCherry* were cultured in methionine drop-out medium, arrested in G1 with α-factor treatment and released into YPAD plus 2 mM methionine, leading to metaphase arrest (due to Cdc20 depletion). At 2 hours after the release, microscopy images were acquired. Spc42 and Nic96 are components of the spindle pole body (SPB) and the nuclear pore complex (NPC), respectively, and SPB signals were much brighter than NPC signals. Images show representative examples where sister *tetO*s are, and are not, separated (two and one CFP dots, respectively). Graphs show % of sister *tetO*s separation during metaphase. n=50–52 for each strain; *p*-values were obtained using Fisher's exact test.

A

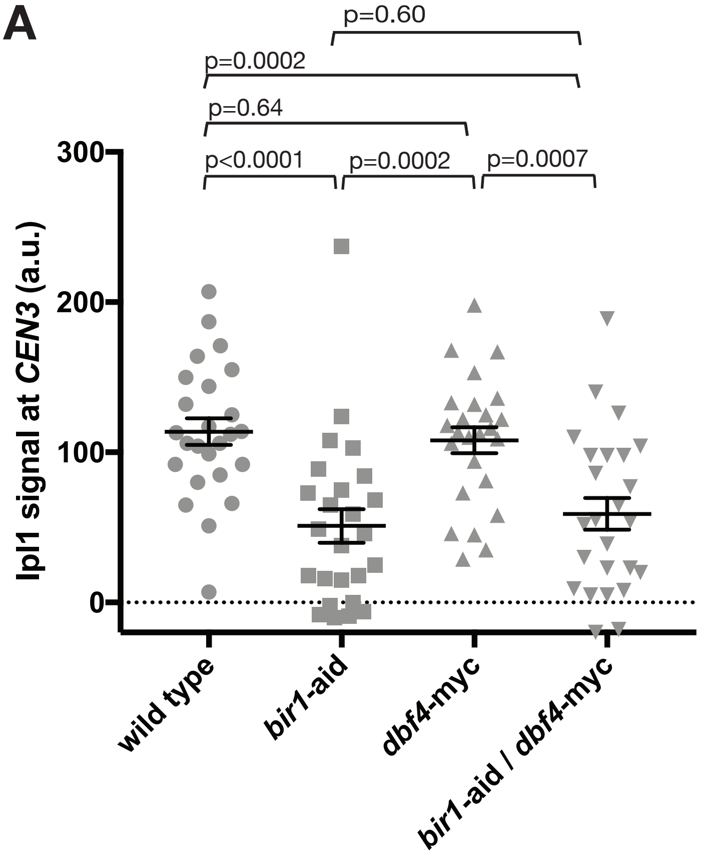

B

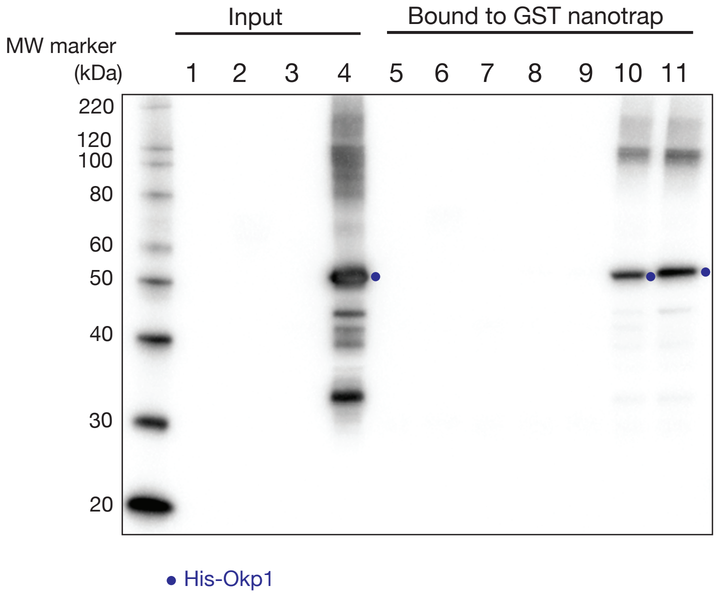

C

	INPUT						PULL-DOWN					
	Lane 2		Lane 3		Lane 4		Lane 9		Lane 10		Lane 11	
	Score	Coverage	Score	Coverage	Score	Coverage	Score	Coverage	Score	Coverage	Score	Coverage
**GST**	**42,125**	93%	**22,124**	92%	3,468	70%	**25,120**	91%	**84,819**	95%	**43,334**	90%
**Sli15 (1-401)**	**79,724**	90%	4,174	65%	3,286	67%	7,308	67%	**141,988**	88%	8,815	70%
**Sli15 (393-698)**	404	45%	**29,749**	89%	590	59%	280	24%	1,030	66%	**37,360**	91%
**Hisx6-Okp1**	63	7%	489	29%	**30,034**	79%	1439	47%	**19,069**	73%	**17,879**	73%
**Ame1**	0	0%	0	0%	**5,977**	72%	200	19%	**3,059**	61%	**3,628**	72%
**Mcm21**	0	0%	90	7%	**6,889**	68%	306	30%	**7,528**	72%	**7,237**	72%
**Ctf19**	0	0%	0	0%	128	14%	0	0%	23	4%	27	4%

(Figure S2, legend on next page)

Figure S2. Effect of *dbf4-myc* on Ipl1 localization at centromeres, and supplemental data for GST pull-down assay. Related to Figure 2.

A. *dbf4-myc* does not affect Ipl1 localization at centromeres in the presence and absence of Bir1. *DBF4*$^{+}$ *BIR1*$^{+}$ (wild-type, T12858), *bir1-aid* (T12860) *dbf4-myc* (T13385) and *bir1-aid dbf4-myc* (T13386) cells with *IPL1-GFP*, *TIR*, *GAL1-10* promoter-*CEN3-tetOs*, *TetR-3×CFP*, *mCherry-TUB1* and *MET3* promoter-*CDC20* were treated and analysed as in Figure 2B and C. Ipl1 signals were quantified at *CEN3* in n=25 cells for each strain. Bars show means and SEMs. *p*-values were obtained using *t*-test.

B. The western blot with an anti-His antibody in Figure 2E is shown in whole.

C. Mass spectrometry analyses of GST pull-down assay. Gel pieces comprising the area between 33 and 75 kDa were excised from Coomassie stained gel in Figure 2E and subjected to mass spectrometry analysis. The data were analyzed with Mascot 2.4.0. The table shows results from lane 2 [Input: GST-Sli15 (1-401)], lane 3 [Input: GST-Sli15 (393-698)], lane 4 [Input: COMA], lane 9 [Pull-down: GST alone + COMA], lane 10 [Pull-down: GST-Sli15 (1-401) + COMA] and lane 11 [Pull-down: GST-Sli15 (393-698) + COMA]. 'Score' represents the probability that the observed match is not a random event [$-10 \times LOG_{10}(P)$, with P = probability that match is random]. 'Coverage' represents the percentage of the protein sequence that is covered by the detected and matched peptides. Scores above the following threshold are indicated in bold: Dependent on background signals, the threshold was set to Score = 10,000 for GST alone, GST-Sli15 (1-401), GST-Sli15 (393-698), and Score = 2,000 for His-Okp1, Ame1, Mcm21 and Ctf19. As most samples contained GST either alone or as a fusion protein, GST was detected with high scores throughout all samples. Lanes 10 and 11 showed a higher abundance of COMA proteins than lane 9, indicating their physical interaction with both GST-Sli15 (1-401) and GST-Sli15 (393-698). Whereas Okp1, Ame1 and Mcm21 were detected with high scores, the score for Ctf19 was reproducibly low. Generally, Ctf19 was hard to detect in mass spectrometry even if it was recovered or expressed in high quantity (e.g. after immuno-precipitation of HA-tagged Ctf19) – perhaps its peptide properties did not allow detection in high sensitivity with mass spectrometry.

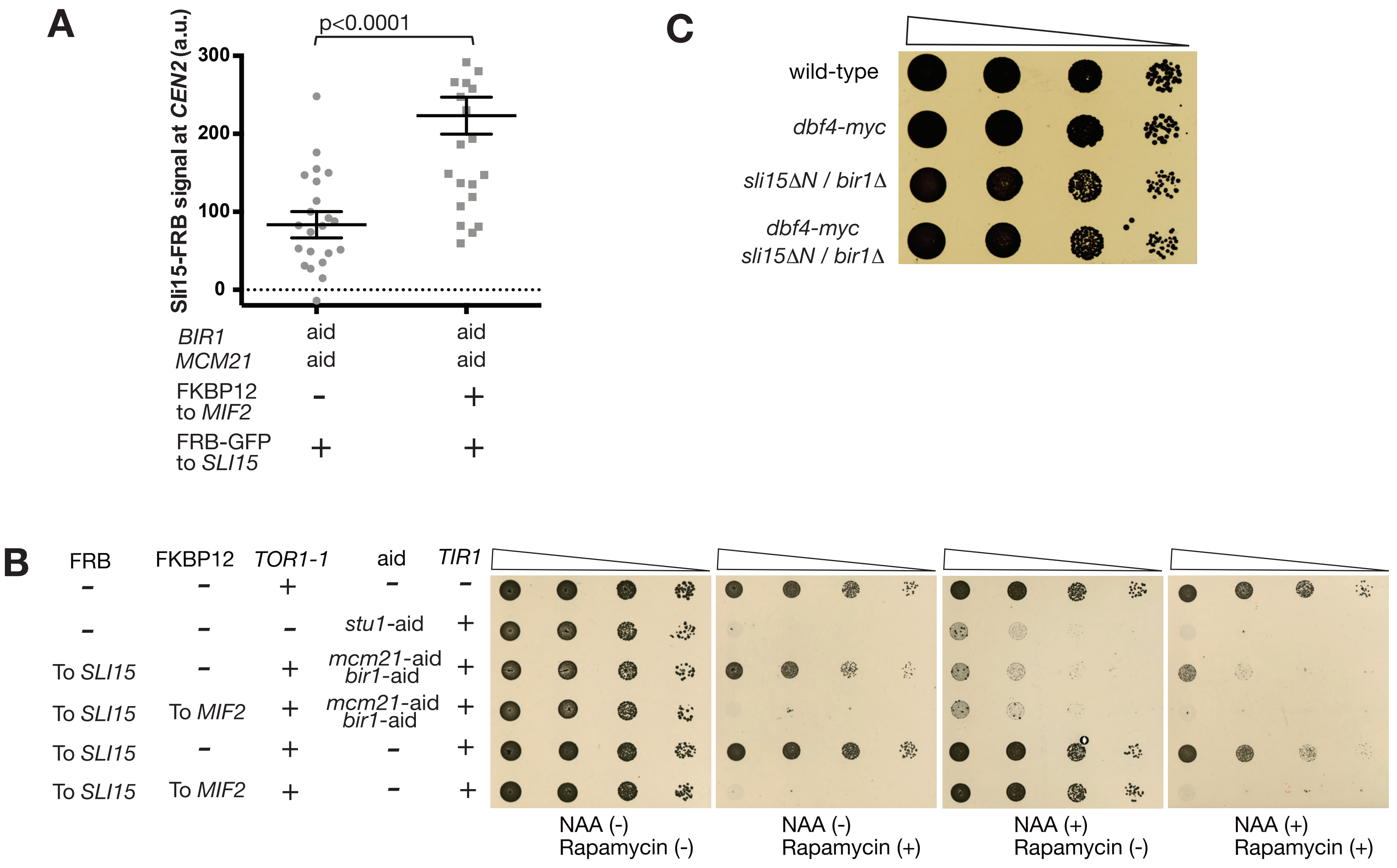

(Figure S3, legend on next page)

Figure S3. Supplemental data for Sli15 tethering to inner kinetochores, and effects of *dbf4-myc* on growth of *bir1*Δ *sli15*ΔN cells. Related to Figure 3 and 4.

A. FKBP12-fused Mif2 recruits FRB-fused Sli15 to the vicinity of *CEN2*. In the experiments shown in Figure 3B, Sli15-FRB signals were quantified at *CEN2* in *bir1-aid mcm21-aid* cells carrying *MIF2* with or without fusion to *FKBP12* (T13444 and T13440, respectively), 30 min after addition of rapamycin. After Z sections were projected to 2D images, Sli15-FRB-GFP signals (colocalising at *CEN2*) were quantified in the area of 2x2 pixels at their maximum intensity. When sister *CEN2* showed separation, Sli15-FRB-GFP signals were averaged between sisters in individual cells. Background was subtracted in every measurement. Bars show means and SEMs. *p*-values were obtained using *t*-test.

B. Tethering Sli15-FRB to Mif2-FKBP12 makes cells inviable. Yeast cells carrying indicated constructs and mutants (the third to sixth strains from the top were the strains used in Figure 3B) were serially diluted (10 time dilution each), spotted on methionine-dropout media with/without NAA and with/without rapamycin (as indicated), and incubated for 3 days. *TOR1-1* cells (T9303) and *stu1*-aid /*TIR* (T10133) were used as controls. We speculate the reason for the lethality of tethering Sli15-FRB to Mif2-FKBP12 as follows: It is known that Ipl1–Sli15 normally leaves centromeres and re-locates to the central spindle during anaphase. We suspect that the presence of Ipl1–Sli15 at the centromere/inner kinetochore during anaphase could cause lethality by disrupting kinetochore–microtubule interaction (since tension is weakened) [S1], reengaging cells to the spindle-assembly checkpoint [S2] and/or delaying the spindle disassembly [S3].

C. *dbf4-myc* does not affect growth of cells with *bir1*Δ *sli15*ΔN. Yeast cells with *bir1*Δ *sli15*ΔN with *DBF4*$^{+}$ or *dbf4-myc*, were serially diluted (10 times dilution each), spotted on plates and incubated for 2 days.

Name	Genotype
PJ69-4A	*MAT* a *trp1-901 leu2-3,112 ura3-52 his3-200 gal4Δ gal80Δ LYS2::GAL1-HIS3 GAL2-ADE2 met2::GAL7-lacZ*
T7107	*MAT* a *ade2-1 trp1-1 leu2-3,112, his311, 15 ura3 can 1-100*
T9030	*MAT* a *ura3::PADH1-OsTIR1-9Myc::URA3*
T9303	*MAT* α *TOR1-1 fpr1Δ::natMX4*
T10133	*MAT* α *ura3::pADH1-OSTIR1-2-9Myc::URA3 stu1-5-aid::clonNAT*
T10141	*MAT* a *cdc20::MET-CDC20::TRP1 ade1::tetR-3CFP::HPH1 SPC42-4mCherry::natMX6 NIC96-4mCherry::natMX6 tetO224 (LEU2) is integrated at +15-kb from CEN12*
T10142	*MAT* a *DBF4-9myc::hphNT1 cdc20::MET-CDC20::TRP1 ade1::tetR-3CFP::HPH1 SPC42-4mCherry::natMX6 NIC96-4mCherry::natMX6 tetO224 (LEU2) is integrated at +15-kb from CEN12*
T12064	*MAT* a *sli15ΔNT* (*Δ2-228)*
T12066	*MAT* a *sli15ΔNT* (*Δ*2-228) *bir1Δ::K.lLEU2*
T12099	*MAT* α *bir1Δ::K.l.LEU2*
T12229	*MAT* a *IPL1-yEGFP::SpHIS5 sl15ΔNT* (*Δ*2-228) *bir1Δ::K.lLEU2 cen15::PGAL-CEN3-tetO2x112::URA3 ade1::tetR-3CFP::HPH1 trp1::PTEF1-mcherry-TUB1::TRP1 cdc20::PMET-CDC20::TRP1*
T12248	*MAT* a *IPL1-yEGFP::SpHIS5 cen15::PGAL-CEN3-tetO2x112::URA3 ade1::tetR-3CFP::HPH1 trp1::PTEF1-mcherry-TUB1::TRP1 cdc20::PMET-CDC20::TRP1*
T12401	*MAT* a *ura3::PADH1-OsTIR1-9Myc::URA3 bir1-aid::clonNAT*
T12410	*MAT* a *ura3::PADH1-OsTIR1-9Myc::URA3 mcm21-aid::clonNAT*
T12466	*MAT* a *sl15ΔNT* (*Δ*2-228) *bir1Δ::K.lLEU2 ura3*::PADH1-OsTIR1-9Myc::*URA3 mcm21*-aid:*clonNAT*
T12467	*MAT* a *sl15ΔNT* (*Δ2-228) ura3::PADH1-OsTIR1-9Myc::URA3 mcm21-aid::clonNAT*
T12613	*MAT* α *bir1Δ::K.l.LEU2 ura3*::*PADH1-OsTIR1-9Myc::URA3 mcm21-aid:clonNAT*
T12614	*MAT* a *bir1-aid*::*clonNAT mcm21-aid*::*clonNAT ura3*::PADH1-*OsTIR1-9Myc::URA3*
T12697	*MAT* a *mcm21-aid::clonNAT ura3::PADH1-OsTIR1-9Myc::URA3 tetO224 (LEU2) is integrated at 0.6-kb from CEN2 ade1::tetR-3CFP::HPH1 SPC42-4mCherry::natMX6*
T12698	*MAT* a *bir1-aid::clonNAT ura3::PADH1-OsTIR1-9Myc::URA3 tetO224 (LEU2) is integrated at 0.6-kb from CEN2 ade1::tetR-3CFP::HPH1 SPC42-4mCherry::natMX6*
T12704	*MAT* a *ura3::PADH1-OsTIR1-9Myc::URA3 tetO224 (LEU2) is integrated at 0.6-kb from CEN2 ade1::tetR-3CFP::HPH1 SPC42-4mCherry::natMX6*
T12714	*MAT* a *bir1*-aid::*clonNAT mcm21*-aid::*clonNAT ura3*::PADH1 *OsTIR1-9Myc::URA3 tetO224 (LEU2) is integrated at 0.6-kb from CEN2 ade1::tetR-3CFP::HPH1 SPC42-4mCherry::natMX6*
T12738	*MAT* a *IPL1-yEGFP::SpHIS5 cen15::PGAL-CEN3-tetO2x112::URA3 ade1::tetR-3CFP::HPH1 trp1::PTEF1-mcherry-TUB1::TRP1 cdc20::PMET-CDC20::TRP1 ura3::PADH1-OsTIR1-9Myc::URA3 mcm21-aid::clonNAT*
T12739	*MAT* a *IPL1-yEGFP::SpHIS5 sl15ΔNT* (*Δ2-228) bir1Δ::K.l.LEU2 cen15::PGAL-CEN3-tetO2x112::URA3 ade1::tetR-3CFP::HPH1 trp1::PTEF1-mcherry-TUB1::TRP1 cdc20::PMET-CDC20::TRP1 ura3::PADH1-OsTIR1-9Myc::URA3 mcm21-aid::clonNAT*
T12809	*MAT* a *DBF4-9Myc::hphNT1 ura3::PADH1-OsTIR1-9Myc::URA3*
T12810	*MAT* alpha *DBF4-9Myc::hphNT1 ura3::PADH1-OsTIR1-9Myc::URA3 bir1-aid::clonNAT*
T12811	*MAT* a *DBF4*-9Myc::*hphNT1 sl15ΔNT* (*Δ*2-228) *bir1Δ::K.lLEU2*
T12858	*MAT* a *IPL1-yEGFP::SpHIS5 cen15::PGAL-CEN3 tetO2x112::URA3 ade1::tetR-3CFP::HPH1 trp1::PTEF1-mcherry-TUB1::TRP1 cdc20::PMET-CDC20::TRP1 ura3::PADH1-OsTIR1-9Myc::URA3*
T12859	*MAT* a *IPL1-yEGFP::SpHIS5 cen15::PGAL-CEN3 tetO2x112::URA3 ade1::tetR-*

	3CFP::HPH1 trp1::PTEF1-mcherry-TUB1::TRP1 cdc20::PMET-CDC20::TRP1 ura3::PADH1-OsTIR1-9Myc::URA3 mcm21-aid::*clonNAT*
T12860	*MAT* a *IPL1-yEGFP::SpHIS5 cen15::PGAL-CEN3-tetO2x112::URA3 ade1::tetR-3CFP::HPH1 trp1::PTEF1-mcherry-TUB1::TRP1 cdc20::PMET-CDC20::TRP1 ura3::PADH1-OsTIR1-9Myc::URA3 bir1-aid*::*clonNAT*
T12861	*MAT* a *IPL1-yEGFP::SpHIS5 cen15::PGAL-CEN3-tetO2x112::URA3 ade1::tetR-3CFP::HPH1 trp1::PTEF1-mcherry-TUB1::TRP1 cdc20::PMET-CDC20::TRP1 ura3::PADH1-OsTIR1-9Myc::URA3 mcm21-aid::clonNAT bir1*-aid::*clonNAT*
T13199	*MAT* a *TOR1-1 fpr1Δ::K.l. LEU2 SLI15-FRB-GFP::KanMX6 cen15::PGAL-CEN3-tetO2x112::URA3 ade1::tetR-3CFP::HPH1 trp1::PTEF1-mcherry-TUB1::TRP1 cdc20::PMET-CDC20::TRP1 ura3::PADH1-OsTIR1-9Myc::URA3*
T13200	*MAT* a *TOR1-1 fpr1Δ::K.l. LEU2 MIF2-2xFKBP12::TRP1 SLI15-FRB-GFP::KanMX6 cen15::PGAL-CEN3-tetO2x112::URA3 ade1::tetR-3CFP::HPH1 trp1::PTEF1-mcherry-TUB1::TRP1 cdc20::PMET-CDC20::TRP1 ura3::PADH1-OsTIR1-9Myc::URA3*
T13201	*MAT* a *TOR1-1 fpr1Δ::K.l. LEU2 SLI15-FRB-GFP::KanMX6 cen15::PGAL-CEN3-tetO2x112::URA3 ade1::tetR-3CFP::HPH1 trp1::PTEF1-mcherry-TUB1::TRP1 cdc20::PMET-CDC20::TRP1 ura3::PADH1-OsTIR1-9Myc::URA3 bir1*-aid*::clonNAT*
T13202	*MAT* a *TOR1-1 fpr1Δ::K.l. LEU2 MIF2-2xFKBP12::TRP1 SLI15-FRB-GFP::KanMX6 cen15::PGAL-CEN3-tetO2x112::URA3 ade1::tetR-3CFP::HPH1 trp1::PTEF1-mcherry-TUB1::TRP1 cdc20::PMET-CDC20::TRP1 ura3::PADH1-OsTIR1-9Myc::URA3 bir1-aid::clonNAT*
T13365	*MAT* a *cdc20::MET-CDC20::TRP1 ade1::tetR-3CFP*::HPH1 mcm21Δ::KanMX4 SPC42-4mCherry::natMX6 NIC96-4mCherry::natMX6 tetO224 (LEU2) is integrated at +15-kb from CEN12*
T13385	*MAT* a *DBF4-9Myc::kanMX4 IPL1-yEGFP::SpHIS5 cen15::PGAL-CEN3-tetO2x112::URA3 ade1::tetR-3CFP::HPH1 trp1::PTEF1-mcherry-TUB1::TRP1 cdc20::PMET-CDC20::TRP1 ura3::PADH1-OsTIR1-9Myc::URA3*
T13386	*MAT* a *DBF4-9Myc::kanMX4 IPL1-yEGFP::SpHIS5 cen15::PGAL-CEN3-tetO2x112::URA3 ade1::tetR-3CFP::HPH1 trp1::PTEF1-mcherry-TUB1::TRP1 cdc20::PMET-CDC20::TRP1 ura3::PADH1-OsTIR1-9Myc::URA3 bir1-aid::clonNAT*
T13438	*MAT* a *TOR1-1 fpr1Δ::K.l. LEU2 SLI15-FRB-GFP::KanMX6 ura3::PADH1-OsTIR1-9Myc::URA3 tetO224 (LEU2) is integrated at 0.6-kb from CEN2 ade1::tetR-3CFP::HPH1 SPC42-4mCherry::natMX6 cdc20::MET-CDC20::TRP1*
T13440	*MAT* a *TOR1-1 fpr1Δ::K.l. LEU2 bir1-aid::clonNAT mcm21-aid::clonNAT SLI15-FRB-GFP::KanMX6 ura3::PADH1-OsTIR1-9Myc::URA3 tetO224 (LEU2) is integrated at 0.6-kb from CEN2 ade1*::tetR-3CFP::HPH1 *SPC42-4mCherry::natMX6 cdc20::MET-CDC20::TRP1*
T13441	*MAT* a *TOR1-1 fpr1Δ::K.l. LEU2 MIF2-2xFKBP12::TRP1 SLI15-FRB-GFP::KanMX6 ura3::PADH1-OsTIR1-9Myc::URA3 tetO224 (LEU2) is integrated at 0.6-kb from CEN2 ade1::tetR-3CFP::HPH1 SPC42-4mCherry::natMX6 cdc20::MET-CDC20::TRP1*
T13444	*MAT* a *TOR1-1 fpr1::K.l. LEU2 MIF2-2xFKBP12::TRP1 bir1-aid::clonNAT mcm21-aid::clonNAT SLI15-FRB-GFP::KanMX6 ura3::PADH1-OsTIR1-9Myc::URA3 tetO224 (LEU2) is integrated at 0.6-kb from CEN2 ade1::tetR-3CFP::HPH1 SPC42-4mCherry::natMX6 cdc20::MET-CDC20::TRP1*

Table S1. The genotypes of budding yeast (*Saccharomyces cerevisiae*) strains used in this study (Related to STAR Methods).
The background of all yeast strains is W303 except for PJ69-4A.

Supplemental References

S1. Tanaka, T.U., Rachidi, N., Janke, C., Pereira, G., Galova, M., Schiebel, E., Stark, M.J., and Nasmyth, K. (2002). Evidence that the Ipl1-Sli15 (Aurora kinase-INCENP) complex promotes chromosome bi-orientation by altering kinetochore-spindle pole connections. Cell *108*, 317-329.

S2. Biggins, S., and Murray, A.W. (2001). The budding yeast protein kinase Ipl1/Aurora allows the absence of tension to activate the spindle checkpoint. Genes & development *15*, 3118-3129.

S3. Buvelot, S., Tatsutani, S.Y., Vermaak, D., and Biggins, S. (2003). The budding yeast Ipl1/Aurora protein kinase regulates mitotic spindle disassembly. The Journal of cell biology *160*, 329-339.
