# Supplementary material for: Aurora B-INCENP Localization at Centromeres/Inner Kinetochores Is Required for Chromosome Bi-orientation in Budding Yeast
Source: Curr Biol. 2019 May 6;29(9):1536–1544.e4. doi: 10.1016/j.cub.2019.03.051 (PMC6509284; doi:10.1016/j.cub.2019.03.051)
Supplement: Document S2. Article plus Supplemental Information [file mmc2.pdf]

Report

Current Biology

Aurora B-INCENP Localization at Centromeres/Inner Kinetochores Is Required for Chromosome Bi-orientation in Budding Yeast

Highlights

- The COMA inner kinetochore sub-complex facilitates chromosome bi-orientation
- COMA physically interacts with Sli15 and recruits Ipl1-Sli15 to the inner kinetochore
- This function of COMA is independent of Bir1 and its role supporting robust cohesion
- Localizing Ipl1-Sli15 at centromeres/inner kinetochores is crucial for bi-orientation

Authors

Luis J. García-Rodríguez, Taciana Kasciukovic, Viola Denninger, Tomoyuki U. Tanaka

Correspondence

t.tanaka@dundee.ac.uk

In Brief

García-Rodríguez et al. show that the COMA inner kinetochore sub-complex physically interacts with Sli15, recruits Ipl1-Sli15 to the inner kinetochore, and promotes chromosome bi-orientation, independently of Bir1, in budding yeast. They conclude that localization of Ipl1-Sli15 at centromeres/inner kinetochores is required for bi-orientation.

García-Rodríguez et al., 2019, Current Biology *29*, 1536–1544
May 6, 2019 © 2019 The Author(s). Published by Elsevier Ltd.
https://doi.org/10.1016/j.cub.2019.03.051

Current Biology
Report

Aurora B-INCENP Localization at Centromeres/Inner Kinetochores Is Required for Chromosome Bi-orientation in Budding Yeast

Luis J. García-Rodríguez,[1] Taciana Kasciukovic,[1,2] Viola Denninger,[1,2] and Tomoyuki U. Tanaka[1,3,*]
[1]Centre for Gene Regulation and Expression, School of Life Sciences, University of Dundee, Dow Street, Dundee DD1 5EH, UK
[2]These authors contributed equally
[3]Lead Contact
*Correspondence: t.tanaka@dundee.ac.uk
https://doi.org/10.1016/j.cub.2019.03.051

SUMMARY

For proper chromosome segregation in mitosis, sister kinetochores must interact with microtubules from opposite spindle poles (chromosome bi-orientation) [1, 2]. To promote bi-orientation, Aurora B kinase disrupts aberrant kinetochore-microtubule interactions [3–6]. It has long been debated how Aurora B halts this action when bi-orientation is established and tension is applied across sister kinetochores. A popular explanation for it is that, upon bi-orientation, sister kinetochores are pulled in opposite directions, stretching the outer kinetochores [7, 8] and moving Aurora B substrates away from Aurora-B-localizing sites at centromeres (spatial separation model) [3, 5, 9]. This model predicts that Aurora B localization at centromeres is required for bi-orientation. However, this notion was challenged by the observation that Bir1 (yeast survivin), which recruits Ipl1-Sli15 (yeast Aurora B-INCENP) to centromeres, can become dispensable for bi-orientation [10]. This raised the possibility that Aurora B localization at centromeres is dispensable for bi-orientation. Alternatively, there might be a Bir1-independent mechanism for recruiting Ipl1-Sli15 to centromeres or inner kinetochores [5, 9]. Here, we show that the COMA inner kinetochore sub-complex physically interacts with Sli15, recruits Ipl1-Sli15 to the inner kinetochore, and promotes chromosome bi-orientation, independently of Bir1, in budding yeast. Moreover, using an engineered recruitment of Ipl1-Sli15 to the inner kinetochore when both Bir1 and COMA are defective, we show that localization of Ipl1-Sli15 at centromeres or inner kinetochores is required for bi-orientation. Our results give important insight into how Aurora B disrupts kinetochore-microtubule interaction in a tension-dependent manner to promote chromosome bi-orientation.

RESULTS AND DISCUSSION

COMA Facilitates Chromosome Bi-orientation, Independently of Bir1 and of Its Role in Supporting Robust Peri-centromere Cohesion

If there were a Bir1-independent mechanism of recruiting Ipl1-Sli15 to centromeres or inner kinetochores, regulators of such a mechanism might show negative genetic interaction with *bir1* mutants. In fact, both *bir1-17* mutant and a *sli15* mutant lacking its Bir1-binding domain showed a negative genetic interaction with *mcm21Δ* and *ctf19Δ* [10, 11]. Mcm21 and Ctf19 are non-essential components of the inner kinetochore sub-complex COMA (Ctf19-Okp1-Mcm21-Ame1) [12]. Another study showed that Sli15 localization at kinetochores was partially diminished in *ame1* mutants [13]. Thus, COMA may recruit Ipl1-Sli15 to inner kinetochores independently of Bir1 to promote bi-orientation and maintain cell viability.

Because *bir1* mutations showed a stronger genetic interaction with *mcm21Δ* than with *ctf19Δ* at physiological culture conditions (20°C and 27°C) [11], we studied the Mcm21 function further. We fused *BIR1* and *MCM21* with the auxin-induced degron tag (*bir1-aid* and *mcm21-aid*) and investigated how this double depletion affected cell growth (Figures 1A and S1A). *STU1* is an essential gene, and *stu1-aid* was used as a control [14]. The *bir1-aid* suppressed cell growth in the presence of auxin, but not as completely as did *stu1-aid*. The *mcm21-aid* did not suppress growth on its own but, when combined with *bir1-aid*, showed further growth suppression than did *bir1-aid* alone (Figure 1A). A similar result was obtained using *bir1Δ* combined with *mcm21-aid* (Figure S1B). Thus, combined depletion (or deletion) of Bir1 and Mcm21 showed a synthetic growth defect.

The COMA complex promotes robust sister chromatid cohesion at peri-centromere regions by recruiting Dbf4-dependent kinase (DDK) to kinetochores [15–18]. Robust peri-centromere cohesion is important for bi-orientation [15, 16, 19]. We addressed whether the above effects of Mcm21 depletion are due to a defect in peri-centromere cohesion. If so, a C-terminus-tagged *DBF4* (*dbf4-myc*), which impairs DDK recruitment to kinetochores [17], should show a similar defect to Mcm21 depletion. In fact, *dbf4-myc* produced a defect in peri-centromere cohesion in similar extent to (or slightly greater than) *mcm21* deletion (Figures S1C and S1D). However, in contrast

 © 2019 The Author(s). Published by Elsevier Ltd.
This is an open access article under the CC BY license (http://creativecommons.org/licenses/by/4.0/).

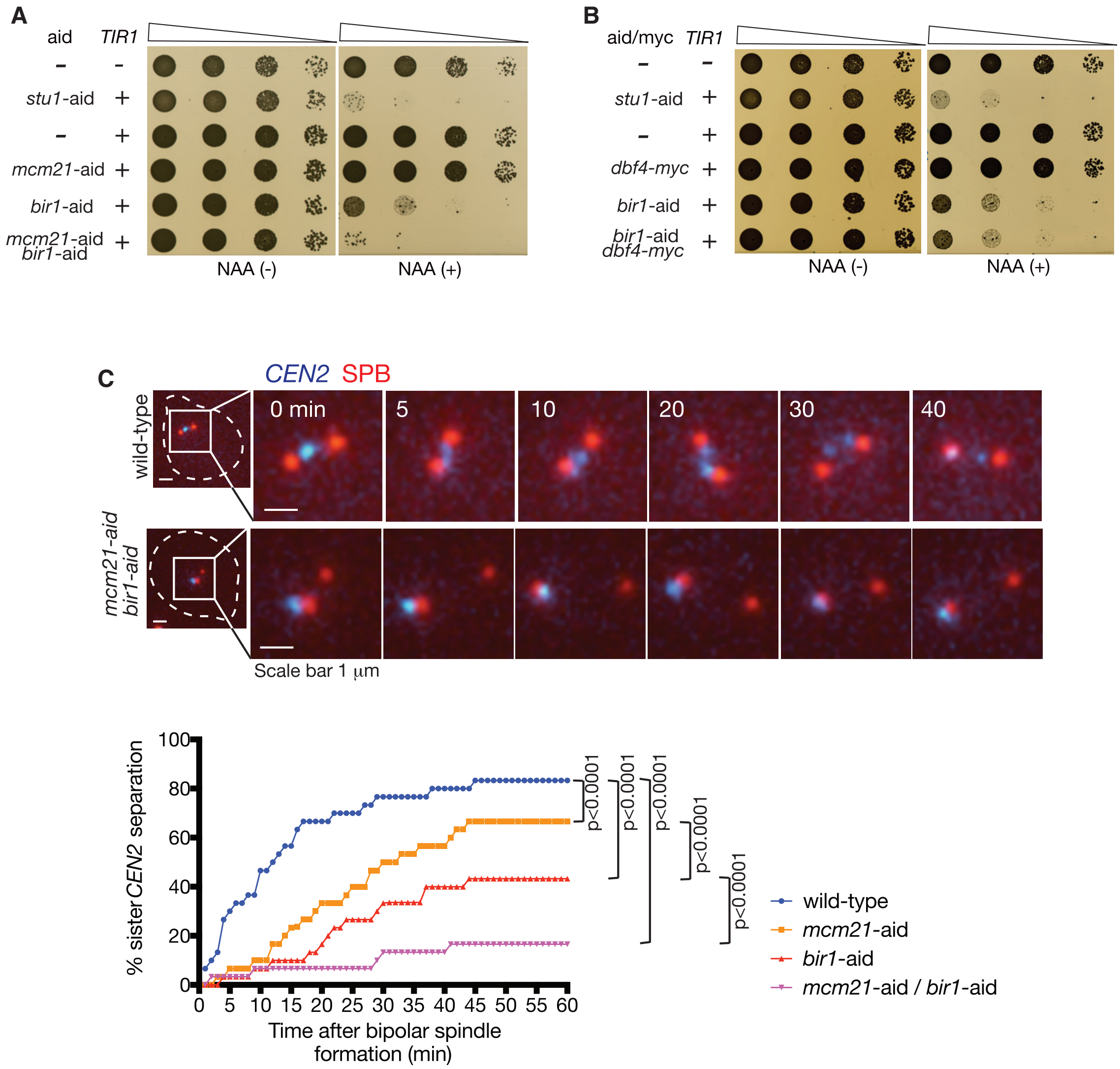

Figure 1. Bir1 and COMA Independently Promote Chromosome Bi-orientation

(A) Bir1 depletion shows synthetic growth defects when combined with Mcm21 depletion. Yeast cells shown here were serially diluted (10 times each), spotted on plates, and incubated for 2 days in the presence (right) and absence (left) of 1-naphthaleneacetic acid (NAA).

(B) Bir1 depletion shows no synthetic growth defects when combined with *dbf4-myc*. Yeast cells shown here were treated and analyzed as in (A).

(C) Bir1 depletion and Mcm21 depletion cause further defects in chromosome bi-orientation when combined. *MCM21*$^+$ *BIR1*$^+$ (wild-type; T12704), *mcm21-aid* (T12697), *bir1-aid* (T12698), and *mcm21-aid bir1-aid* (T12714) cells with *TIR*, *CEN2-tetOs*, *TetR-3×CFP*, and *SPC42-4×mCherry* were arrested in G1 with α-factor treatment and released into fresh medium. NAA was added 30 min before the release and also upon release. Microscopy images were acquired from 25 min after the release for 90 min at 1-min intervals. x axis shows time relative to separation of spindle pole bodies (SPBs) (Spc42-mCherry), which is defined as time 0. y axis shows % of cells showing separation of sister *CEN2s* on the bipolar spindle (i.e., after SPB separation) for at least two consecutive time points at or prior to indicated time points. n = 30 for each strain; p values were obtained using Kolmogorov-Smirnov test.

See also Figure S1.

to Mcm21 depletion, *dbf4-myc* showed no synthetic growth defect with Bir1 depletion (Figure 1B). This suggests that the synthetic growth defect of Mcm21-depletion and Bir1-depletion is not due to a defect in peri-centromere cohesion.

We analyzed the efficiency of chromosome bi-orientation establishment in wild-type control, individual Bir1 and Mcm21 depletions, and the double depletion. To assay this, we visualized a chosen centromere (*CEN2*) and spindle poles in live-cell fluorescence microscopy, scored the percentage of cells with bi-orientation (separation of sister *CEN2*), and plotted against time after formation of a bipolar spindle (Figure 1C). In a wild-type control, bi-orientation was established in the majority of cells within 15 min. Individual Mcm21 and Bir1 depletions showed moderate and substantial delays, respectively, in bi-orientation establishment. Intriguingly, Mcm21 and Bir1 double depletion showed a further delay in bi-orientation than did individual depletions. In the double depletion, only ~17% of cells showed bi-orientation after 45 min. The effects of Mcm21 and

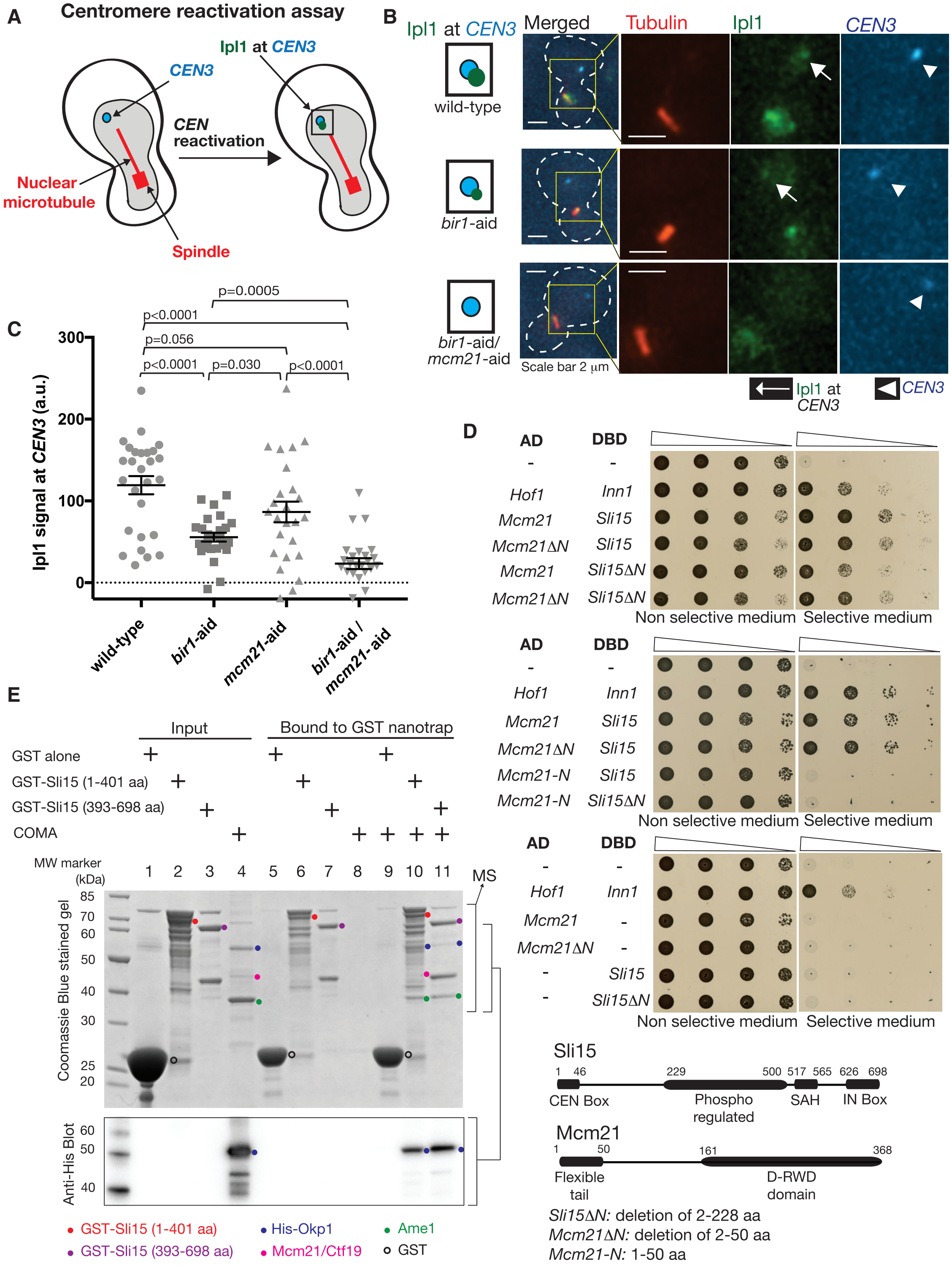

Figure 2. COMA Physically Interacts with Sli15 and Recruits Ipl1-Sli15 to the Inner Kinetochore Independently of Bir1

(A) Diagram shows the method of analyzing Ipl1 signals at isolated *CEN3*. *CEN3* under *GAL1-10* promoter was inactivated by transcription from the promoter, which prevented interaction with microtubules and placed it away from the spindle (left) [23, 24]. After reactivation of *CEN3* (by shutting off the promoter) during metaphase arrest, Ipl1 at *CEN3* was evaluated (right). See more details in STAR Methods.

(B and C) Bir1 and COMA independently recruit Ipl1 to centromeres. *MCM21*$^{+}$ *BIR1*$^{+}$ (wild-type; T12858), *mcm21-aid* (T12859), *bir1-aid* (T12860), and *mcm21-aid bir1-aid* (T12861) cells with *IPL1-GFP*, *TIR*, *GAL1-10* promoter-*CEN3-tetOs*, *TetR-3×CFP*, *mCherry-TUB1*, and *MET3* promoter-*CDC20* were treated and analyzed as in diagram in (A). Immediately after *CEN3* was reactivated, images were acquired for 10 min with a 1-min interval: (B) shows representative images of

(legend continued on next page)

Bir1 depletions seemed to be additive after 45 min, suggesting that COMA and Bir1 independently facilitate chromosome bi-orientation.

Note that, whereas essential COMA components Okp1-Ame1 facilitate outer kinetochore assembly, non-essential components Ctf19-Mcm21 have little such function [20–22]. It is therefore unlikely that the bi-orientation delay in Mcm21 depletion was due to reduced ability of the kinetochore for interacting with microtubules. Consistent with this, *CEN2* was always located in the vicinity of one spindle pole (i.e., interacted with microtubules) when bi-orientation was defective due to Mcm21 depletion or Mcm21 and Bir1 double depletion (Figure 1C, image, bottom).

COMA Physically Interacts with Sli15 and Recruits Ipl1-Sli15 to the Inner Kinetochore, Independently of Bir1

Independent roles of COMA and Bir1 in facilitating chromosome bi-orientation may be due to their independent functions of recruiting Ipl1-Sli15 to centromeres or inner kinetochores. To address this possibility, we analyzed the localization of Ipl1 at centromeres. Because centromeres locate on the mitotic spindle, it was difficult to distinguish between Ipl1 localization on centromeres and on spindle microtubules. Therefore, to analyze Ipl1 localization specifically at a centromere, we isolated a chosen centromere (*CEN3*) from the spindle by inactivating it and thereby inhibiting kinetochore-microtubule interactions [23, 24] (Figure 2A, left). Subsequently, we reactivated *CEN3*, allowing its recapture by spindle microtubules (centromere reactivation assay). We analyzed Ipl1 localization at *CEN3* after reactivation but before recapture by spindle microtubules (Figures 2A, right, and 2B).

The level of Ipl1 signals at *CEN3* was quantified in wild-type control, individual Bir1 and Mcm21 depletions, and the double depletion (Figure 2C). The Ipl1 localization was marginally reduced by Mcm21 depletion and more clearly reduced by Bir1 depletion. Intriguingly, Mcm21 and Bir1 double depletion showed greater reduction of Ipl1 localization at *CEN3* compared with individual depletions. The effect of Mcm21 and Bir1 depletions on Ipl1 localization at *CEN3* seemed to be additive, suggesting that COMA and Bir1 independently promote Ipl1 localization at centromeres or kinetochores.

We also investigated the effect of *dbf4-myc*, which impairs peri-centromere cohesion [17], on Ipl1 localization on *CEN3* (Figure S2A). *DBF4* wild-type and *dbf4-myc* showed a similar level of Ipl1 localization at *CEN3*. Bir1 depletion reduced Ipl1 localization at *CEN3*, but this reduction was similar when combined with *DBF4* wild-type and *dbf4-myc*. These results with *dbf4-myc* contrast with those with Mcm21 depletion (Figure 2C), suggesting that reduced Ipl1 localization at centromeres (or kinetochores) with Mcm21 depletion in the absence of Bir1 was not due to weakened peri-centromere cohesion.

How then does COMA promote Ipl1 localization at centromeres or kinetochores, independently of Bir1? COMA may physically interact with Ipl1-Sli15 to enable their recruitment to the inner kinetochore, and we investigated possible physical interactions between Mcm21 and Sli15 using the yeast two-hybrid method (Figure 2D). Indeed, Mcm21 and Sli15 showed physical interaction, and this was not dependent on the Bir1-binding domain of Sli15 (Sli15 N terminus 1–228 amino acids [aa]) [28, 29] or the flexible Mcm21 N terminus (1–50 aa) [26].

We also addressed whether Sli15 directly interacts with COMA, using purified recombinant proteins. Glutathione S-transferase (GST)-Sli15 (1–401 aa), GST-Sli15 (393–698 aa), and COMA were separately expressed in bacteria and purified (Figure 2E, top, lanes 2–4). Both of the immobilized GST-Sli15 (1–401 aa) and GST-Sli15 (393–698 aa) pulled down COMA components, as analyzed by Coomassie blue staining (Figure 2E, top, lanes 5–11), western blots (Figures 2E, bottom, and S2B) and mass spectrometry (Figure S2C). Thus, COMA physically and directly interacts with Sli15, independently of Bir1, to recruit Ipl1-Sli15 to the inner kinetochore.

Engineered Recruitment of Ipl1-Sli15 to the Inner Kinetochore Restores Bi-orientation when Both COMA and Bir1 Are Defective

Our results suggest that the level of Ipl1 localization at centromeres is correlated well with efficiency of chromosome bi-orientation when Bir1 and Mcm21 were depleted individually and in combination (Figures 1C and 2C). In particular, with Bir1 and Mcm21 double depletion, both bi-orientation and Ipl1 localization at centromeres (or inner kinetochores) were almost completely abolished. This raises the possibility that Ipl1-Sli15 localization at centromeres or inner kinetochores is crucial for chromosome bi-orientation. One way to test this is to engineer recruitment of Ipl1-Sli15 to centromeres or inner kinetochores in the absence of Bir1 and Mcm21 and to test whether this can rescue bi-orientation.

To engineer Ipl1-Sli15 recruitment to inner kinetochores, we used the rapamycin-dependent association between FRB and FKBP12 [30] and fused FRB and FKBP12 to Sli15 and Mif2, respectively. Mif2 is the yeast CENP-C ortholog and an inner

wild-type (top); Bir1-depleted (middle); and Bir1- and Mcm21-depleted (bottom) cells. Graph in (C) shows Ipl1 signals quantified at *CEN3* in n = 25–27 cells for each strain. Bars show means and SEMs. p values were obtained using t test.

(D) Mcm21 and Sli15 interact in the yeast two-hybrid assay. The indicated constructs were fused to the Gal4 transcriptional activation domain (AD) and Gal4 DNA-binding domain (DBD). If the AD- and DBD-fused constructs physically interact, yeast cells grow on plates with selective medium. Yeast cells (10 times serial dilution) were incubated at 30°C for 2 days. Hof1 and Inn1 were used as a positive control [25]. Diagram shows domain structures of Sli15 and Mcm21 [26, 27].

(E) Recombinant COMA components are pulled down by immobilized recombinant Sli15. The following samples were run on the SDS-PAGE and stained with Coomassie blue (top): GST alone, indicated GST fusion proteins and COMA components (including His-tagged Okp1) were expressed in, and purified from, *E. coli* cells (lanes 1–4). GST and the GST fusion proteins were immobilized on GST Nanotrap; purified COMA components were added, washed, and proteins bound to GST Nanotrap were analyzed (lanes 9–11). Lanes 5–8 show controls. A bracket, marked "MS," shows the area for mass spectrometry analyses (Figure S2C). A western blot (from a separate SDS-PAGE running the same samples) with anti-His antibody (to detect His1-Okp1) is shown below; connected brackets on the right show corresponding areas. Figure S2B shows the whole western blot. We interpret that colored dots indicate the proteins listed at bottom. The GST fusion proteins showed some truncation or degradation, and only their full-length bands are marked by colored dots.

See also Figure S2.

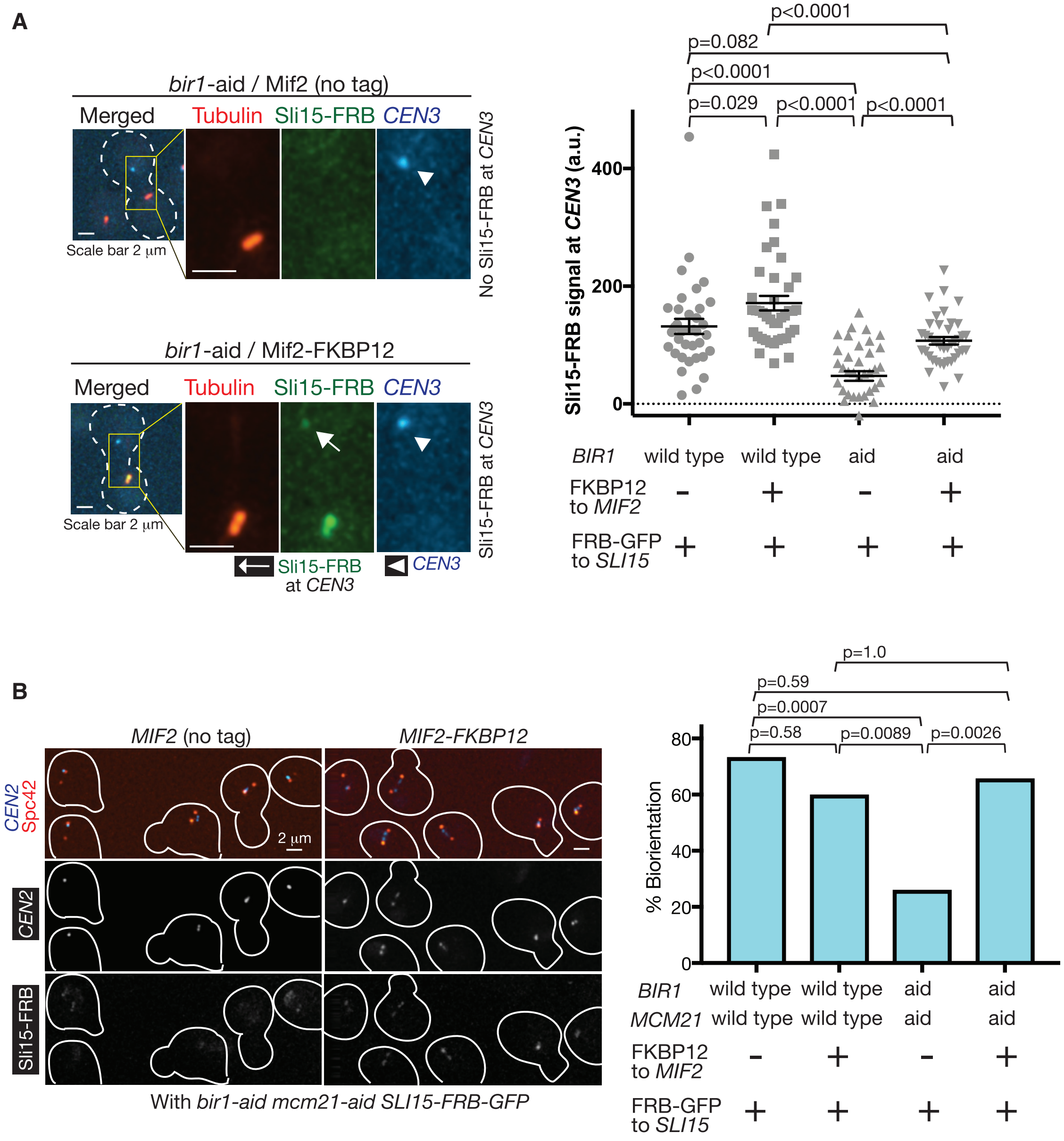

Figure 3. Engineered Recruitment of Sli15 to the Inner Kinetochore Restores Bi-orientation when Both COMA and Bir1 Are Defective

(A) FKBP12-fused Mif2 recruits FRB-fused Sli15 to isolated *CEN3*. (a) *BIR1+* (wild-type) or *bir1-aid* and (b) *MIF2* with or without fusion to *FKBP12* were combined as indicated below the graph (T13199–T13202 from left to right). All strains carried *SLI15-FRB-GFP*, *TIR*, *TOR1-1, fpr1Δ, GAL1-10* promoter-*CEN3-tetOs*, *TetR-3×CFP*, *mCherry-TUB1*, and *MET3* promoter-*CDC20.* Cells were treated as in Figure 2B, except that rapamycin was added 30 min before the start of image acquisition. Microscope images (left) show representative examples of *bir1-aid MIF2* (no tag; top) and *bir1-aid MIF2-FKBP12* (bottom) cells. Sli15-FRB signals were quantified at *CEN3* in n = 26–30 cells for each strain (graph at right). Bars show means and SEMs. p values were obtained by t test.

(B) Engineered Sli15 association with Mif2 restores bi-orientation when both COMA and Bir1 are defective. (a) *BIR1*$^{+}$ *MCM21*$^{+}$ (wild-type) or *bir1-aid mcm21-aid* and (b) *MIF2* with or without fusion to *FKBP12* were combined as indicated below the graph (T13438, T13441, T13440, and T13444 from left to right). All strains carried *SLI15-FRB-GFP*, *TIR*, *TOR1-1, fpr1Δ*, *CEN2-tetOs*, *TetR-3×CFP*, *SPC42-4×mCherry*, and *MET3* promoter-*CDC20.* They were cultured in methionine drop-out medium, arrested in G1 with α-factor treatment, and released into YPAD plus 2 mM methionine, leading to metaphase arrest (due to Cdc20 depletion). At 2 h following the release, microscopy images were acquired. Rapamycin was added 30 min before the start of image acquisition. Representative images are shown on left; cell shapes are shown in white lines. y axis of the graph (right) shows % of sister *CEN2* separation, representing its bi-orientation. n = 30–35 for each strain; p values were obtained using Fisher's exact test.

See also Figures S3A and S3B.

kinetochore component and was chosen for this purpose because its inner kinetochore localization would not be affected by Mcm21 depletion [22]. As in Figure 2A, we isolated *CEN3* from the mitotic spindle and studied Sli15-FRB localization at *CEN3* in the presence of rapamycin (Figure 3A). When Mif2 was not fused to FKBP12 (control), Bir1 depletion considerably reduced the

level of Sli15-FRB at *CEN3*, consistently with Figure 2B. Fusion of FKBP12 to Mif2 rescued Sli15-FRB localization at *CEN3* to an almost normal level, when Bir1 was depleted (Figure 3A). Thus, indeed, Sli15 is recruited to the inner kinetochore by this engineered system.

We next evaluated efficiency of chromosome bi-orientation with the engineered recruitment of Sli15 to inner kinetochores. As in Figure 3A, FRB and FKBP12 were fused to Sli15 and Mif2, respectively, and bi-orientation frequency was evaluated by visualizing *CEN2* on the spindle in the presence of rapamycin. When Mif2 was not fused to FKBP12 (control), very little Sli15-FRB co-localized with *CEN2* (Figures 3B, image, left, and S3A), and Bir1 and Mcm21 double depletion significantly reduced frequency of bi-orientation (Figure 3B, graph; as also shown in Figure 1C). Importantly, in the Bir1 and Mcm21 double depletion, fusion of FKBP12 to Mif2 enhanced Sli15-FRB co-localization with *CEN2* (Figures 3B, image, right, and S3A) and rescued frequency of bi-orientation to an almost normal level (Figure 3B, graph). Thus, engineered recruitment of Sli15 to inner kinetochores rescued bi-orientation when both Bir1 and COMA were defective. Intriguingly, the engineered Sli15 recruitment made cells inviable even with wild-type Bir1 and Mcm21, which we speculate is due to abnormally sustained localization of Ipl1-Sli15 at inner kinetochores during anaphase (Figure S3B). Overall, the above results suggest that Ipl1-Sli15 localization at centromeres or inner kinetochores is crucial for chromosome bi-orientation.

Ipl1 Still Localizes at the Inner Kinetochore with *bir1*Δ *sli15*ΔN, which Is Dependent on COMA

It was previously reported that, when Sli15 lacks its Bir1-binding domain 2–228 aa (*sli15*ΔN), yeast cells still establish bi-orientation and grow almost normally in the absence of Bir1 [10]. Based on this, it was proposed that Ipl1-Sli15 localization at centromeres is dispensable for chromosome bi-orientation [10]. Given our finding that COMA promotes recruitment of Ipl1-Sli15 to inner kinetochores independently of Bir1 (Figure 2B), bi-orientation and cell growth in *bir1*Δ *sli15*ΔN may be dependent on COMA. Consistent with this, cell growth of *bir1*Δ *sli15*ΔN cells was severely reduced when combined with Mcm21 depletion (Figure 4A). In contrast, growth of *bir1*Δ *sli15*ΔN cells was not affected when combined with *dbf4-myc* (Figure S3C), which showed a defect in peri-centromere cohesion (Figure S1D). Therefore, the effect of Mcm21 depletion on the growth of *bir1*Δ *sli15*ΔN-cells was not due to weakened peri-centromere cohesion.

We next examined localization of Ipl1 on *CEN3*, which was isolated from the spindle as in Figure 2A. Intriguingly, with *bir1*Δ *sli15*ΔN, Ipl1 localization at *CEN3* was similar to that with the wild-type control (Figure 4B). This result contrasts with Ipl1 localization at *CEN3* with Bir1 depletion alone, cells of which showed considerable reduction of Ipl1 there (Figure 2B). This suggests that *sli15*ΔN enhances Ipl1 localization at centromeres in the absence of Bir1, though the mechanism for this is still unclear. In any case, when combined with Mcm21 depletion, the *bir1*Δ *sli15*ΔN cells showed a very severe defect in Ipl1 localization at *CEN3* (Figure 4B). In conclusion, Ipl1 still localizes at the inner kinetochore with *bir1*Δ *sli15*ΔN, and this localization depends on COMA.

Implications of This Study

In this study, we have demonstrated that the COMA kinetochore sub-complex physically interacts with Sli15 and recruits Ipl1-Sli15 to the inner kinetochore, independently of Bir1. Physical interactions between Sli15 and COMA components (Ctf19 and Mcm21) have also been shown in a recently posted preprint, using chemical crosslinking of recombinant proteins [31]. Furthermore, using an engineered system for recruiting Ipl1-Sli15 to the inner kinetochore when both Bir1 and COMA are defective, we were able to show that localization of Ipl1-Sli15 at centromeres or inner kinetochores is crucial for bi-orientation in budding yeast. We presume that the centromere and inner kinetochore are suitable locations of Ipl1-Sli15 to enable efficient phosphorylation of outer kinetochores that drives error correction.

Our finding also gives insight into how tension across sister kinetochores halts the Aurora B action of disrupting kinetochore-microtubule interaction. One popular model for this is the spatial separation model, i.e., tension causes kinetochore stretching, which moves outer kinetochore substrates away from Aurora-B-localizing sites at centromeres [3, 5, 9]. Consistent with this model, Aurora B delocalizes from outer kinetochores when bi-orientation is established in budding yeast [3, 32], outer kinetochores (but not centromeres) are under differential regulation by Aurora B activity before and after establishment of bi-orientation in human cells [33], and outer kinetochore components are dephosphorylated when tension is applied [34, 35]. The spatial separation model predicts that Aurora B localization at centromeres is required for bi-orientation. However, it has been shown that bi-orientation can be established in the absence of Bir1 in budding yeast [10], and this has raised the possibility that Ipl1 (Aurora B) localization at centromeres is dispensable for bi-orientation—if so, that might rule out the spatial separation model. Our results suggest that the COMA-dependent mechanism still recruits Ipl1 (Aurora B) to the inner kinetochore in the absence of Bir1, at least partially compensating for its centromere localization and supporting bi-orientation. If our conclusion is correct, the spatial separation model still remains a plausible model for Aurora-B-driven error correction, at least, in budding yeast.

On the other hand, our data do not exclude other models explaining how tension halts the Aurora B action of disrupting kinetochore-microtubule interaction. For example, the kinetochore-microtubule interface may form a stable structure by itself when tension is applied [36], which may overcome the Aurora B action. Localization of Aurora B at the outer kinetochore may have crucial roles in rendering its action tension dependent [37, 38]. Aurora B kinase activity or counteracting phosphatase activity may be directly regulated by tension [39, 40]. Our finding that Ipl1 (Aurora B) localization at the centromere or inner kinetochore is crucial for bi-orientation may also shed new light on these models.

In this study, we used budding yeast as a model organism. Are the conclusions in this study also the case in vertebrate cells? Ipl1, Sli15, Bir1, and COMA are conserved from yeast to vertebrates, and their vertebrate counterparts are called Aurora B, INCENP, survivin, and CENP-O/P/Q/U, respectively [41, 42]. The complex consisting of Aurora B, INCENP, survivin, and Borealin (Nbl1 in yeast) is called the chromosomal

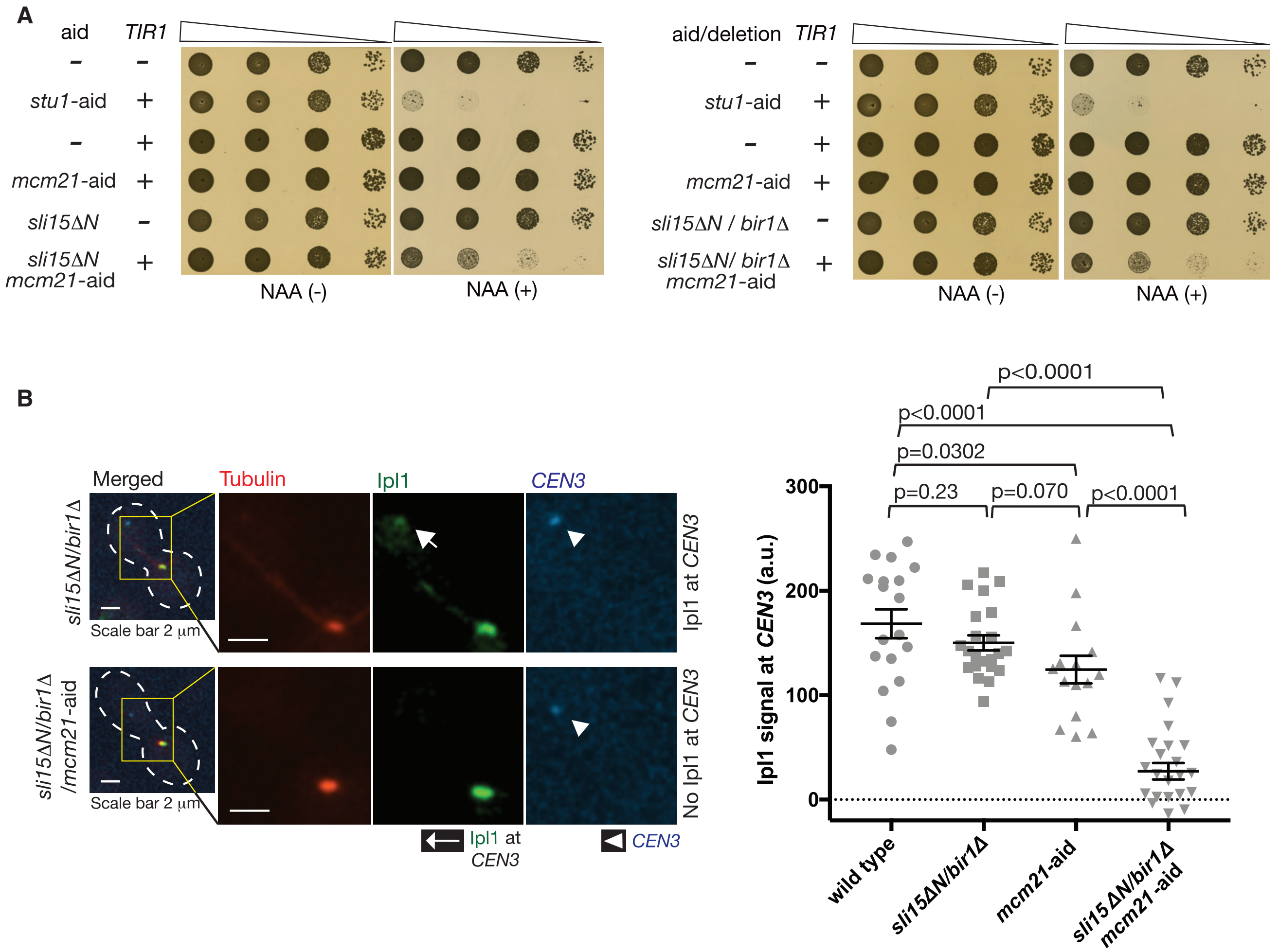

Figure 4. Ipl1 Still Localizes at the Inner Kinetochore with *bir1*Δ *sli15*ΔN, and This Is Dependent on COMA

(A) *bir1*Δ *sli15*ΔN (*sli15*ΔN; deletion of 2–228 aa) shows synthetic growth defects when combined with Mcm21 depletion. Yeast cells shown here were serially diluted (10 times each), spotted on plates, and incubated for 2 days in the presence (right) and absence (left) of NAA.

(B) Ipl1 localizes at the inner kinetochore with *bir1*Δ *sli15*ΔN dependent on COMA. *BIR1*$^+$ *SLI15*$^+$ *MCM21*$^+$ (wild-type; T12248), *bir1*Δ *sli15*Δ*N* (T12229), *mcm21-aid TIR* (T12738), and *bir1*Δ *sli15*Δ*N mcm21-aid TIR* (T12739) cells with *IPL1-GFP*, *GAL1-10* promoter-*CEN3-tetOs*, *TetR-3×CFP*, *mCherry-TUB1*, and *MET3* promoter-*CDC20* were treated and analyzed as in Figure 2B. Immediately after reactivation of *CEN3*, images were acquired for 10 min with a 1-min interval. Representative images (left) of T12229 and T12739 cells are shown at top and bottom, respectively. Ipl1 signals were quantified at *CEN3* in n = 15–25 cells for each strain (graph at right). Bars show means and SEMs. p values were obtained using t test.

See also Figure S3C.

passenger complex [43]. Crucially, it was recently reported that INCENP lacking its survivin-binding domain (N terminus) still supports bi-orientation in human cells [44], as does yeast Sli15 lacking its N terminus [10]. If mechanisms were conserved between yeast and vertebrate cells, CENP-O/P/Q/R would promote recruitment of Aurora B-INCENP to inner kinetochores independently of survivin. Intriguingly, in the *Xenopus* egg extract system, the inner kinetochore was not fully assembled when INCENP lacked its N terminus (presumably, in contrast to human cells); in this circumstance, this INCENP mutant could no longer support bi-orientation, even if the outer kinetochore assembly seemed normal [45]. Therefore, inner kinetochore components may indeed support Aurora B-INCENP localization and error correction, independently of survivin, in vertebrate cells.

STAR★METHODS

Detailed methods are provided in the online version of this paper and include the following:

- KEY RESOURCES TABLE
- CONTACT FOR REAGENT AND RESOURCE SHARING
- EXPERIMENTAL MODEL AND SUBJECT DETAILS
  - Yeast strains and cell culture
  - Depletion of AID-tagged proteins
  - Engineered association between proteins
- METHOD DETAILS
  - Microscopy image acquisition
  - Centromere reactivation assay
  - Yeast two-hybrid assay

- ○ Purification of GST-Sli15 (1-401 aa) and GST-Sli15 (393-698 aa)
- ○ Purification of recombinant COMA
- ○ GST pull-down assay
- ○ Mass spectrometry analysis of pull-down samples
- ● QUANTIFICATION AND STATISTICAL ANALYSIS

SUPPLEMENTAL INFORMATION

Supplemental Information can be found online at https://doi.org/10.1016/j.cub.2019.03.051.

ACKNOWLEDGMENTS

We thank Tanaka lab members, F. Herzog, and A. Desai for discussion; M.J.R. Stark and F. Herzog for sharing unpublished results; Z. Yue for technical help; L. Clayton for editing the manuscript; Dundee Imaging Facility for help in microscopy; Dundee Proteomic Facility for help in mass spectrometry; and K. Bloom, R. Ciosk, P. James, J.E. Haber, M. Kanemaki, K. Labib, U.K. Laemmli, K. Nasmyth, K.E. Sawin, A. Straight, R.Y. Tsien, EUROSCARF, and Yeast Resource Centre for reagents. This work was supported by an ERC advanced grant (322682) and the Wellcome Trust (096535/Z/11/Z and 097945/Z/11/Z). T.U.T. is a Wellcome Trust Principal Research Fellow.

AUTHOR CONTRIBUTIONS

Conceptualization, T.U.T. and L.J.G.-R.; Methodology, L.J.G.-R., T.K., and V.D.; Investigation, L.J.G.-R., T.K., and V.D.; Formal Analysis, L.J.G.-R., T.K., and V.D.; Visualization, L.J.G.-R. and T.U.T.; Writing, T.U.T., L.J.G.-R., T.K., and V.D.; Supervision, T.U.T.; Project Administration, T.U.T.; Funding Acquisition, T.U.T.

DECLARATION OF INTERESTS

The authors declare no competing interests.

Received: January 9, 2019
Revised: March 6, 2019
Accepted: March 22, 2019
Published: April 18, 2019

REFERENCES

1. Tanaka, T.U. (2010). Kinetochore-microtubule interactions: steps towards bi-orientation. EMBO J. *29*, 4070–4082.
2. Lampson, M.A., and Grishchuk, E.L. (2017). Mechanisms to avoid and correct erroneous kinetochore-microtubule attachments. Biology (Basel) *6*, 1–18.
3. Tanaka, T.U., Rachidi, N., Janke, C., Pereira, G., Galova, M., Schiebel, E., Stark, M.J., and Nasmyth, K. (2002). Evidence that the Ipl1-Sli15 (Aurora kinase-INCENP) complex promotes chromosome bi-orientation by altering kinetochore-spindle pole connections. Cell *108*, 317–329.
4. Kelly, A.E., and Funabiki, H. (2009). Correcting aberrant kinetochore microtubule attachments: an Aurora B-centric view. Curr. Opin. Cell Biol. *21*, 51–58.
5. Krenn, V., and Musacchio, A. (2015). The Aurora B kinase in chromosome bi-orientation and spindle checkpoint signaling. Front. Oncol. *5*, 225.
6. Kalantzaki, M., Kitamura, E., Zhang, T., Mino, A., Novák, B., and Tanaka, T.U. (2015). Kinetochore-microtubule error correction is driven by differentially regulated interaction modes. Nat. Cell Biol. *17*, 421–433.
7. Uchida, K.S., Takagaki, K., Kumada, K., Hirayama, Y., Noda, T., and Hirota, T. (2009). Kinetochore stretching inactivates the spindle assembly checkpoint. J. Cell Biol. *184*, 383–390.
8. Maresca, T.J., and Salmon, E.D. (2009). Intrakinetochore stretch is associated with changes in kinetochore phosphorylation and spindle assembly checkpoint activity. J. Cell Biol. *184*, 373–381.
9. Tanaka, T.U., Clayton, L., and Natsume, T. (2013). Three wise centromere functions: see no error, hear no break, speak no delay. EMBO Rep. *14*, 1073–1083.
10. Campbell, C.S., and Desai, A. (2013). Tension sensing by Aurora B kinase is independent of survivin-based centromere localization. Nature *497*, 118–121.
11. Makrantoni, V., Ciesiolka, A., Lawless, C., Fernius, J., Marston, A., Lydall, D., and Stark, M.J.R. (2017). A functional link between Bir1 and the *Saccharomyces cerevisiae* Ctf19 kinetochore complex revealed through quantitative fitness analysis. G3 (Bethesda) *7*, 3203–3215.
12. De Wulf, P., McAinsh, A.D., and Sorger, P.K. (2003). Hierarchical assembly of the budding yeast kinetochore from multiple subcomplexes. Genes Dev. *17*, 2902–2921.
13. Knockleby, J., and Vogel, J. (2009). The COMA complex is required for Sli15/INCENP-mediated correction of defective kinetochore attachments. Cell Cycle *8*, 2570–2577.
14. Vasileva, V., Gierlinski, M., Yue, Z., O'Reilly, N., Kitamura, E., and Tanaka, T.U. (2017). Molecular mechanisms facilitating the initial kinetochore encounter with spindle microtubules. J. Cell Biol. *216*, 1609–1622.
15. Fernius, J., and Marston, A.L. (2009). Establishment of cohesion at the pericentromere by the Ctf19 kinetochore subcomplex and the replication fork-associated factor, Csm3. PLoS Genet. *5*, e1000629.
16. Ng, T.M., Waples, W.G., Lavoie, B.D., and Biggins, S. (2009). Pericentromeric sister chromatid cohesion promotes kinetochore biorientation. Mol. Biol. Cell *20*, 3818–3827.
17. Natsume, T., Müller, C.A., Katou, Y., Retkute, R., Gierliński, M., Araki, H., Blow, J.J., Shirahige, K., Nieduszynski, C.A., and Tanaka, T.U. (2013). Kinetochores coordinate pericentromeric cohesion and early DNA replication by Cdc7-Dbf4 kinase recruitment. Mol. Cell *50*, 661–674.
18. Hinshaw, S.M., Makrantoni, V., Harrison, S.C., and Marston, A.L. (2017). The kinetochore receptor for the cohesin loading complex. Cell *171*, 72–84.e13.
19. Tanaka, T., Fuchs, J., Loidl, J., and Nasmyth, K. (2000). Cohesin ensures bipolar attachment of microtubules to sister centromeres and resists their precocious separation. Nat. Cell Biol. *2*, 492–499.
20. Mehta, G.D., Agarwal, M., and Ghosh, S.K. (2014). Functional characterization of kinetochore protein, Ctf19 in meiosis I: an implication of differential impact of Ctf19 on the assembly of mitotic and meiotic kinetochores in Saccharomyces cerevisiae. Mol. Microbiol. *91*, 1179–1199.
21. Hornung, P., Troc, P., Malvezzi, F., Maier, M., Demianova, Z., Zimniak, T., Litos, G., Lampert, F., Schleiffer, A., Brunner, M., et al. (2014). A cooperative mechanism drives budding yeast kinetochore assembly downstream of CENP-A. J. Cell Biol. *206*, 509–524.
22. Schmitzberger, F., Richter, M.M., Gordiyenko, Y., Robinson, C.V., Dadlez, M., and Westermann, S. (2017). Molecular basis for inner kinetochore configuration through RWD domain-peptide interactions. EMBO J. *36*, 3458–3482.
23. Tanaka, K., Mukae, N., Dewar, H., van Breugel, M., James, E.K., Prescott, A.R., Antony, C., and Tanaka, T.U. (2005). Molecular mechanisms of kinetochore capture by spindle microtubules. Nature *434*, 987–994.
24. Tanaka, K., Kitamura, E., and Tanaka, T.U. (2010). Live-cell analysis of kinetochore-microtubule interaction in budding yeast. Methods *51*, 206–213.
25. García-Rodríguez, L.J., De Piccoli, G., Marchesi, V., Jones, R.C., Edmondson, R.D., and Labib, K. (2015). A conserved Polε binding module in Ctf18-RFC is required for S-phase checkpoint activation downstream of Mec1. Nucleic Acids Res. *43*, 8830–8838.
26. Schmitzberger, F., and Harrison, S.C. (2012). RWD domain: a recurring module in kinetochore architecture shown by a Ctf19-Mcm21 complex structure. EMBO Rep. *13*, 216–222.

27. Fink, S., Turnbull, K., Desai, A., and Campbell, C.S. (2017). An engineered minimal chromosomal passenger complex reveals a role for INCENP/Sli15 spindle association in chromosome biorientation. J. Cell Biol. *216*, 911–923.
28. Jeyaprakash, A.A., Klein, U.R., Lindner, D., Ebert, J., Nigg, E.A., and Conti, E. (2007). Structure of a survivin-Borealin-INCENP core complex reveals how chromosomal passengers travel together. Cell *131*, 271–285.
29. Nakajima, Y., Tyers, R.G., Wong, C.C., Yates, J.R., 3rd, Drubin, D.G., and Barnes, G. (2009). Nbl1p: a Borealin/Dasra/CSC-1-like protein essential for Aurora/Ipl1 complex function and integrity in Saccharomyces cerevisiae. Mol. Biol. Cell *20*, 1772–1784.
30. Chen, J., Zheng, X.F., Brown, E.J., and Schreiber, S.L. (1995). Identification of an 11-kDa FKBP12-rapamycin-binding domain within the 289-kDa FKBP12-rapamycin-associated protein and characterization of a critical serine residue. Proc. Natl. Acad. Sci. USA *92*, 4947–4951.
31. Fischboeck, J., Singh, S., Potocnjak, M., Hagemann, G., Solis, V., Woike, S., Ghodgaonkar, M., Andreani, J., and Herzog, F. (2018). The COMA complex is required for positioning Ipl1 activity proximal to Cse4 nucleosomes in budding yeast. bioRxiv. https://doi.org/10.1101/444570.
32. Shimogawa, M.M., Widlund, P.O., Riffle, M., Ess, M., and Davis, T.N. (2009). Bir1 is required for the tension checkpoint. Mol. Biol. Cell *20*, 915–923.
33. Liu, D., Vader, G., Vromans, M.J., Lampson, M.A., and Lens, S.M. (2009). Sensing chromosome bi-orientation by spatial separation of aurora B kinase from kinetochore substrates. Science *323*, 1350–1353.
34. Keating, P., Rachidi, N., Tanaka, T.U., and Stark, M.J. (2009). Ipl1-dependent phosphorylation of Dam1 is reduced by tension applied on kinetochores. J. Cell Sci. *122*, 4375–4382.
35. Welburn, J.P., Vleugel, M., Liu, D., Yates, J.R., 3rd, Lampson, M.A., Fukagawa, T., and Cheeseman, I.M. (2010). Aurora B phosphorylates spatially distinct targets to differentially regulate the kinetochore-microtubule interface. Mol. Cell *38*, 383–392.
36. Akiyoshi, B., Sarangapani, K.K., Powers, A.F., Nelson, C.R., Reichow, S.L., Arellano-Santoyo, H., Gonen, T., Ranish, J.A., Asbury, C.L., and Biggins, S. (2010). Tension directly stabilizes reconstituted kinetochore-microtubule attachments. Nature *468*, 576–579.
37. DeLuca, K.F., Lens, S.M., and DeLuca, J.G. (2011). Temporal changes in Hec1 phosphorylation control kinetochore-microtubule attachment stability during mitosis. J. Cell Sci. *124*, 622–634.
38. Caldas, G.V., DeLuca, K.F., and DeLuca, J.G. (2013). KNL1 facilitates phosphorylation of outer kinetochore proteins by promoting Aurora B kinase activity. J. Cell Biol. *203*, 957–969.
39. Sandall, S., Severin, F., McLeod, I.X., Yates, J.R., 3rd, Oegema, K., Hyman, A., and Desai, A. (2006). A Bir1-Sli15 complex connects centromeres to microtubules and is required to sense kinetochore tension. Cell *127*, 1179–1191.
40. Gelens, L., Qian, J., Bollen, M., and Saurin, A.T. (2018). The importance of kinase-phosphatase integration: lessons from mitosis. Trends Cell Biol. *28*, 6–21.
41. Schleiffer, A., Maier, M., Litos, G., Lampert, F., Hornung, P., Mechtler, K., and Westermann, S. (2012). CENP-T proteins are conserved centromere receptors of the Ndc80 complex. Nat. Cell Biol. *14*, 604–613.
42. Biggins, S. (2013). The composition, functions, and regulation of the budding yeast kinetochore. Genetics *194*, 817–846.
43. Carmena, M., Wheelock, M., Funabiki, H., and Earnshaw, W.C. (2012). The chromosomal passenger complex (CPC): from easy rider to the godfather of mitosis. Nat. Rev. Mol. Cell Biol. *13*, 789–803.
44. Hengeveld, R.C.C., Vromans, M.J.M., Vleugel, M., Hadders, M.A., and Lens, S.M.A. (2017). Inner centromere localization of the CPC maintains centromere cohesion and allows mitotic checkpoint silencing. Nat. Commun. *8*, 15542.
45. Haase, J., Bonner, M.K., Halas, H., and Kelly, A.E. (2017). Distinct roles of the chromosomal passenger complex in the detection of and response to errors in kinetochore-microtubule attachment. Dev. Cell *42*, 640–654.e5.
46. James, P., Halladay, J., and Craig, E.A. (1996). Genomic libraries and a host strain designed for highly efficient two-hybrid selection in yeast. Genetics *144*, 1425–1436.
47. Amberg, D.C., Burke, D.J., and Strathern, J.N. (2005). Methods in Yeast Genetics: A Cold Spring Harbor Laboratory Course Manual (Cold Spring Harbor Laboratory).
48. Tanaka, K., Kitamura, E., Kitamura, Y., and Tanaka, T.U. (2007). Molecular mechanisms of microtubule-dependent kinetochore transport toward spindle poles. J. Cell Biol. *178*, 269–281.
49. Michaelis, C., Ciosk, R., and Nasmyth, K. (1997). Cohesins: chromosomal proteins that prevent premature separation of sister chromatids. Cell *91*, 35–45.
50. Hill, A., and Bloom, K. (1987). Genetic manipulation of centromere function. Mol. Cell. Biol. *7*, 2397–2405.
51. Bressan, D.A., Vazquez, J., and Haber, J.E. (2004). Mating type-dependent constraints on the mobility of the left arm of yeast chromosome III. J. Cell Biol. *164*, 361–371.
52. Uhlmann, F., Wernic, D., Poupart, M.A., Koonin, E.V., and Nasmyth, K. (2000). Cleavage of cohesin by the CD clan protease separin triggers anaphase in yeast. Cell *103*, 375–386.
53. Gandhi, S.R., Gierliński, M., Mino, A., Tanaka, K., Kitamura, E., Clayton, L., and Tanaka, T.U. (2011). Kinetochore-dependent microtubule rescue ensures their efficient and sustained interactions in early mitosis. Dev. Cell *21*, 920–933.
54. Li, S., Yue, Z., and Tanaka, T.U. (2017). Smc3 deacetylation by Hos1 facilitates efficient dissolution of sister chromatid cohesion during early anaphase. Mol. Cell *68*, 605–614.e4.
55. Janke, C., Magiera, M.M., Rathfelder, N., Taxis, C., Reber, S., Maekawa, H., Moreno-Borchart, A., Doenges, G., Schwob, E., Schiebel, E., and Knop, M. (2004). A versatile toolbox for PCR-based tagging of yeast genes: new fluorescent proteins, more markers and promoter substitution cassettes. Yeast *21*, 947–962.
56. Sheff, M.A., and Thorn, K.S. (2004). Optimized cassettes for fluorescent protein tagging in Saccharomyces cerevisiae. Yeast *21*, 661–670.
57. Nishimura, K., Fukagawa, T., Takisawa, H., Kakimoto, T., and Kanemaki, M. (2009). An auxin-based degron system for the rapid depletion of proteins in nonplant cells. Nat. Methods *6*, 917–922.
58. Haruki, H., Nishikawa, J., and Laemmli, U.K. (2008). The anchor-away technique: rapid, conditional establishment of yeast mutant phenotypes. Mol. Cell *31*, 925–932.

STAR★METHODS

KEY RESOURCES TABLE

REAGENT or RESOURCE	SOURCE	IDENTIFIER
Antibodies		
Anti-AID antibody, sheep polyclonal	Karim Labib lab	N/A
Anti-6His tag antibody, mouse monoclonal (HIS.H8)	abcam	ab18184
Anti-Sheep IgG (whole molecule)–Peroxidase antibody produced in donkey	Sigma Aldrich	A3415
Goat anti-mouse IgG H&L, HRP	abcam	ab97023
Bacterial and Virus Strains		
One Shot TOP10 Chemically Competent Cells	Invitrogen	C404003
Chemicals, Peptides, and Recombinant Proteins		
1- Naphthaleneacetic acid (NAA)	Sigma-Aldrich	N0640-25G
α-factor	Pepceuticals Ltd	N/A
Rapamycin	LC Laboratories	R-5000
GST-Trap Agarose	ChromoTek	sta-10
HisPur Nic-NTA Superflow agarose	ThermoScientific	25214
Glutathione Sepharose 4 Fast Flow	Sigma	GE17-5132-01
Concanavalin A	Sigma-Aldrich	C7275
Experimental Models: Organisms/Strains		
S. cerevisiae W303 and its derivatives (see Table S1 for detail)	This study	See Table S1
PJ69-4A	Philip James	See [46]
Recombinant DNA		
pGADT7 (*LEU2* marker)	Clontech Takara	630442
pGBKT7 (*TRP1* marker)	Clontech Takara	630443
pGADT7-*HOF1* (*LEU2* marker)	Karim Labib lab	pKL772
pGBKT7-*INN1* (*TRP1* marker)	Karim Labib lab	pKL769
pGADT7-*MCM21* (*LEU2* marker)	This study	pT3294
pGADT7-*MCM21ΔN* (*LEU2* marker)	This study	pT3295
pGADT7-*MCM21−N* (*LEU2* marker)	This study	pT3296
pGBKT7-*SLI15* (*TRP1* marker)	This study	pT3297
pGBKT7-*SLI15ΔN* (*TRP1* marker)	This study	pT3298
pRS405-*SLI15ΔN* (*URA3* marker)	This study	pT3299
pGEX6P-1-*GST-SLI15* (1-401)	This study	pT3176
pGEX6P-1-*GST-SLI15* (393-698)	This study	pT3177
pETDuet-*His-OKP1-AME1*	This study	pT3299
pRSFDuet-*CTF19-MCM21*	This study	pT3333
Software and Algorithms		
Volocity 6.1.1	PerkinElmer	http://www.perkinelmer.com/
Prism 6.0	Graph-Pad	https://www.graphpad.com/
ApE A plasmid editor	M. Wayne Davis	http://jorgensen.biology.utah.edu/wayned/ape/
SoftWoRx 6.5.2	GE Healthcare	http://incelldownload.gehealthcare.com/bin/download_data/SoftWoRx/6.5.2/SoftWoRx.htm
Mascot 2.6	Matrix Science	http://www.matrixscience.com
ImageLab 4.1	Bio-Rad	http://www.bio-rad.com/en-uk/product/image-lab-software?ID=KRE6P5E8Z
Other		
Glass-bottom dish	MatTek	P35G-1.5-10-C P35G-1.5-14-C

CONTACT FOR REAGENT AND RESOURCE SHARING

Further information and requests for resources and reagents should be directed to and will be fulfilled by the Lead Contact, Tomoyuki Tanaka (t.tanaka@dundee.ac.uk).

EXPERIMENTAL MODEL AND SUBJECT DETAILS

Yeast strains and cell culture

The background of yeast strains (W303) and the methods for yeast culture have been described previously [47, 48]. To synchronize cells in the cell cycle, yeast cells were arrested in G1 phase by treatment with yeast mating pheromone (α-factor) and subsequently released to fresh media [47]. Cells were cultured at 25°C in YPA medium containing 2% glucose (YPAD) unless otherwise stated. Constructs of *GAL1-10* promoter-*CEN3-tetOs* [23, 49, 50], *TetR-3×CFP* [49, 51], *MET3* promoter-*CDC20* [52], *mCherry-TUB1* [53], *SPC42-4×mCherry*, *NIC96-4×mCherry* [54], *CEN2-tetO*s (*tetO×224* was inserted 600 bp away from *CEN2*) [17] and *tetO*s at 15 kb from *CEN12* [17] were described previously. To generate *bir1Δ* and *mcm21Δ*, the whole coding region of the gene was replaced with the *Kl. LEU2* and *KAN-MX4* cassette, respectively, using a one-step PCR procedure [55], i.e., the selection marker was amplified by PCR primers carrying additional 50-mer single-strand DNAs that are homologous to 5′ and 3′ regions of the gene, and introduced into yeast cells by transfection, followed by selection of cells with targeted gene deletion. *IPL1* was tagged with yEGFP at its C terminus at its original locus using the yEGFP-SpHIS5 cassette (pKT128) as a PCR template using a one-step PCR procedure [56], i.e., *yEGFP* and the selection marker were amplified by PCR primers carrying additional 50-mer single-strand DNAs that are homologous to the end of the coding region and 3′ region of the gene, and introduced into yeast cells by transfection, followed by selection of cells with the *IPL1-yEGFP* fusion gene. *sli15*ΔN (deletion of 2-228 aa) replaced the original *SLI15*+ wild-type gene at its original locus, using the loop-in and loop-out strategy, as follows; 1) *SLI15* promoter, the ATG start codon and *SLI15* coding sequence corresponding to 229–465 aa were cloned into pRS405 vector (URA3 marker) (pT3299), 2) the construct was integrated (loop-in) at *SLI15* locus by homologous recombination after cutting at the *NruI* site within the *SLI15* promoter, 3) the strain was grown with 5-FOA to remove the *URA3* marker (loop-out) by homologous recombination, and 4) strains were checked by PCR and DNA sequencing to select those with *sli15*ΔN (deletion of 2-228 aa) and without the original $SLI15^{+}$ wild-type gene. The genotype of strains used in this study is provided in Table S1. The strain numbers that are not included in Figure legends are as follows: Figure 1A (T7107, T10133, T9030, T12410, T12401 and T12614 from top to bottom), Figure 1B (T7107, T10133, T9030, T12809, T12401 and T12810 from top to bottom), Figure 4A left (T7107, T10133, T9030, T12410, T12064 and T12467 from top to bottom), Figure 4A right (T7107, T10133, T9030, T12410, T12066 and T12466 from top to bottom), Figure S1B (T7107, T10133, T9030, T12410, T12099 and T12613 from top to bottom) and Figure S3C (T9030, T12809, T12066 and T12811 from top to bottom).

Depletion of AID-tagged proteins

To deplete Bir1 and Mcm21, *BIR1 and MCM21* were fused to an *aid* tag (auxin-inducible degron tag) at their C-termini at the original loci in the strain carrying the rice F-box gene *TIR1* [57]. In the presence of auxin NAA (1-Naphthaleneacetic acid; 2 mM on plates and 0.5 mM in liquid media, unless otherwise stated), *aid*-tagged proteins interact with the SCF E3 ubiquitin ligase, mediated by Tir1, which leads to their ubiquitylation and degradation by the proteasome [57]. To detect Bir1-aid and Mcm21-aid in western blots, an anti-AID tag sheep antibody (generated by MRC PPU Reagents and Services, University of Dundee, and was kindly provided by Karim Labib) was used.

Engineered association between proteins

To engineer association between Sli15 and Mif2, *SLI15* was tagged with *FRB-GFP* using a *FRB-GFP-kanMX6* (pFA6a-FRB-GFP-kanMX6) cassette, and *MIF2* with *2×FKBP12-TRP1* cassette (pFA6a-2×FKBP12-TRP1) at their C-termini at their original gene loci by a one-step PCR method [58]. For this experiment, yeast strains also carried *TOR1-1*, which conferred rapamycin resistance, and *fpr1Δ* mutations. Association of *FRB* and *FKBP12* fusion proteins was induced by addition of 1 μM of rapamycin to culture media.

METHOD DETAILS

Microscopy image acquisition

During time-lapse imaging, yeast cells were immobilized on a glass-bottomed dish (MatTek, P35G-1.5-10-C) coated with concanavalin A (Sigma C7275), and maintained in synthetic-complete (SC) plus YPA medium (3:1 ratio) [24, 47]. For imaging during metaphase arrest of cells with *MET3* promoter-*CDC20, 2* mM methionine was added to the medium to ensure Cdc20 depletion. Where relevant, NAA was added to the medium during imaging to maintain protein degradation, and Rapamycin was added to the medium during imaging to maintain FRB–FKBP12 interaction. Images were acquired using a DeltaVision Elite microscope (Applied Precision), an UPlanSApo 100 × objective lens (Olympus; NA 1.40), SoftWoRx software (Applied Precision), and a CoolSnap HQ (Photometrics). We acquired 7–11 (0.7 μm apart) z sections, which were subsequently processed through deconvolution, and analyzed with Volocity (Improvision) software. CFP, GFP, and mCherry signals were discriminated using the 89006 multi-band filter set (Chroma). For the image panels in Figures, Z sections were projected to two-dimensional images.

Centromere reactivation assay

To analyze Ipl1 or Sli15 localization at a centromere isolated from the spindle, the centromere re-activation assay was used [23, 24]. In this assay, kinetochore assembly was delayed on a chosen centromere by transcription from the *GAL* promoter (*GAL1-10* promoter-*CEN3-tetOs* replacing *CEN15* on chromosome *XV*). This increased the distance between the centromere and the mitotic spindle, allowing observation of protein localization specifically at *CEN3* after inducing kinetochore assembly on the centromere by turning off the *GAL* promoter in metaphase-arrested cells. Cells with *GAL1-10* promoter-*CEN3-tetOs* and *MET3* promoter-*CDC20* were cultured overnight in methionine drop-out media with 2% raffinose, treated with α-factor for 2.5 hours (to arrest in G1 phase), and released to fresh media with 2% raffinose, 2% galactose and 2 mM methionine (for Cdc20 depletion and *CEN3* inactivation). After 2 hours, cells were suspended in SC medium containing 2% glucose and methionine to reactivate *CEN3*. Protein localization was analyzed at *CEN3* after *CEN3* reactivation and before *CEN3* interaction with microtubules extended from the spindle. After Z sections were projected to 2D images, Ipl1-GFP and Sli15-FRB-GFP signals (colocalizing at *CEN3*) were quantified in the area of 2x2 pixels at their maximum intensity, using the Voxel Spy tools of Volocity. Background was subtracted in every measurement.

Yeast two-hybrid assay

Two-Hybrid analysis was carried out as in [25]. Briefly, the assay was based on the Gal4 transcription factor and performed after co-transformation of derivatives of pGADT7 (Gal4 activation domain; *LEU2* marker; Clontech) and pGBKT7 (Gal4 DNA binding domain; *TRP1* marker; Clontech) into the yeast strain PJ69-4A (two-hybrid strain [46]). For each assay, independent colonies from the transformation were mixed together in water and spotted in ten-fold dilutions onto SC medium lacking tryptophan and leucine (selective for pGADT7 and pGBKT7, but non-selective for the two-hybrid interaction) and SC medium lacking tryptophan, leucine, histidine and adenine (selective for the two-hybrid interaction).

Purification of GST-Sli15 (1-401 aa) and GST-Sli15 (393-698 aa)

SLI15 coding DNAs, corresponding to 1–401 aa and 393-698 aa, were fused with GST on pGEX6P-1 (GE Healthcare), which were named pT3176 and pT3177, respectively. The pT3176 and pT3177 were introduced into RosettaGami2 *E. coli* cells (Novagen). The *E. coli* cells were grown in LB medium, and the GST fusion protein was expressed at 18°C with 0.1 mM IPTG induction overnight. Cells were harvested and disrupted using an Emulsiflex cell disruptor (ATA Scientific) in the lysis buffer (50 mM HEPES-NaOH pH7.6, 1 M NaCl, 5 mM β-mercaptoethanol, 1% Triton X-100) supplemented with cOmplete protease inhibitors (Roche). The cleared lysate was loaded onto 1ml GSTrap FF column (GE Healthcare). Subsequently the proteins, trapped on the column, were eluted with 40 mM reduced glutathione (Sigma) in the elution buffer (50 mM HEPES-NaOH pH7.6, 0.3 M NaCl, 1 mM DTT, 0.05% NP-40, 10% glycerol). The eluate was loaded onto Superdex 200 10/300 column (GE Healthcare) equilibrated with 50 mM HEPES-NaOH pH7.6, 0.3 M NaCl, 1 mM DTT, 0.05% NP-40, 15% glycerol, and fractions containing full-length GST-Sli15 (1–401 aa) and GST-Sli15 (393-698 aa) were pooled and concentrated.

Purification of recombinant COMA

DNA fragments coding for *His-OKP1* and *AME1* were cloned into the pETDuet (Amp) vector (Novagen), while coding regions for *CTF19* and *MCM21* were cloned into the pRSFDuet (Kan) vector (Novagen). The two constructs (pT3329 and pT3333, respectively) were introduced together into RosettaGami2(DE3)pLysS *E. coli* cells (Novagen), and protein expression was induced with 0.2 mM IPTG at 16°C overnight. Cells were lysed in buffer 50 mM Tris-HCl pH 7.5, 250 mM NaCl, 0.5% Igepal CA-630, 10 mM β-glycerophosphate, 5% glycerol, 10 mM imidazole and 1 mM PMSF, supplemented with cOmplete protease inhibitors (Roche). The cleared lysate was incubated with His-Pur Nic-NTA Superflow agarose (Thermo) for 1.5 h at 4°C. Subsequently, the agarose beads were washed with 5CV wash buffer (50 mM Tris-HCl pH 7.5, 250 mM NaCl, 0.5% Igepal CA-630 and 20 mM imidazole). The proteins bound on the agarose were eluted in buffer containing 50 mM Tris-HCl pH 7.5, 100 mM NaCl, 0.1% Triton X-100, 1 mM β-mercaptoethanol, 250 mM imidazole and 1 mM PMSF, supplemented with cOmplete protease inhibitors (Roche). Elution fractions were pooled and further purified by gel filtration chromatography (HiLoad 16/60 Superdex 200, GE Healthcare). The gel filtration buffer contained 20 mM HEPES-NaOH pH7.5, 300 mM NaCl, 10% glycerol, 1mM DTT and 1 mM PMSF. The presence of His-Okp1, Ame1, Ctf19 and Mcm21 in the elution peak was confirmed by mass spectrometry (MS/MS) after trypsin digestion, and by western blot with anti-His antibody (Abcam).

GST pull-down assay

GST alone, GST-Sli15 (1-401) or GST-Sli15 (393-698) were incubated together with purified COMA in buffer containing 50 mM HEPES-NaOH pH 7.4, 150 mM NaCl, 5% glycerol, 0.05% Igepal, 2 mM DTT and 0.5 mM EDTA for 20 min at 4°C. This was added to GST-Trap Agarose (sta-10, ChromoTek) and pre-equilibrated with the above buffer. After 1-hour incubation at 4°C with rotation, the beads were washed three times with the above buffer. For elution of the bound proteins, beads were boiled in 2x SDS-PAGE sample buffer. Eluted proteins were separated by SDS–PAGE (Novex 10% Bis-Tris gel), and stained with Coomassie Blue (Imperial Blue, ThermoScientific). We confirmed that the band with the expected size of His-Okp1, visible on the Coomassie-stained gel (Figure 2E, lane 4, 10 and 11), indeed contained His-Okp1 using western blot with anti-His antibody (Abcam). Two SDS-PAGEs ran the same samples (in the same amount) in parallel; one was used for the western blot as mentioned above while the other was used for mass spectrometry analyses (see next section).

Mass spectrometry analysis of pull-down samples

After Coomassie staining, gel pieces were excised from lanes 2, 3, 4, 9, 10 and 11, comprising the expected protein sizes (33 - 75kDa) to cover GST-Sli15 (1-401) (72 kDa), GST-Sli15 (393-698) (60 kDa), His-Okp1 (49 kDa), Mcm21 (43 kDa), Ctf19 (43 kDa) and Ame1 (37 kDa). After destaining the gel pieces, proteins were reduced with 10 mM DTT for 45min at 55°C and alkylated with 55 mM iodoacetamide for 30min at ambient temperature. Digestion was performed with mass-spectrometry grade trypsin (Pierce) at 37°C, once for 4 h, then overnight. Subsequently, peptides were extracted by two rounds of sonication in 50%ACN in 0.1%TFA and one round of sonication in 70%ACN in 0.1%TFA. Pooled extracts of each sample were dried completely, re-suspended in the appropriate solvent and subjected to mass spectrometry. Measurement was performed using a Q Exactive HF, and data were analyzed with Mascot 2.4.0.

QUANTIFICATION AND STATISTICAL ANALYSIS

All the experiments were repeated at least twice and similar results were obtained. Statistical analyses were carried out using Prism software (Graphpad). Methods of statistic tests are stated in each relevant figure legend. Kolmogorov–Smirnov test was used in Figure 1C to test if efficiency in bi-orientation establishment is different between two groups. t test was used in Figures 2C, 3A, 4B, S2A, and S3A to test if there is a significant difference between the means of two groups. Fisher's exact test was used in Figures 3B and S1D to test if frequency of bi-orientation (Figure 3B) and sister separation (Figure S1D) is significantly different between two groups. The null hypotheses in these tests were that the samples were collected randomly and independently from the same population. All p values were two-tailed, and the null hypotheses were reasonably discarded when p values were < 0.05. Sample numbers are also stated in figure legends.

Current Biology, Volume *29*

Supplemental Information

Aurora B-INCENP Localization at Centromeres/Inner Kinetochores Is Required for Chromosome Bi-orientation in Budding Yeast

Luis J. García-Rodríguez, Taciana Kasciukovic, Viola Denninger, and Tomoyuki U. Tanaka

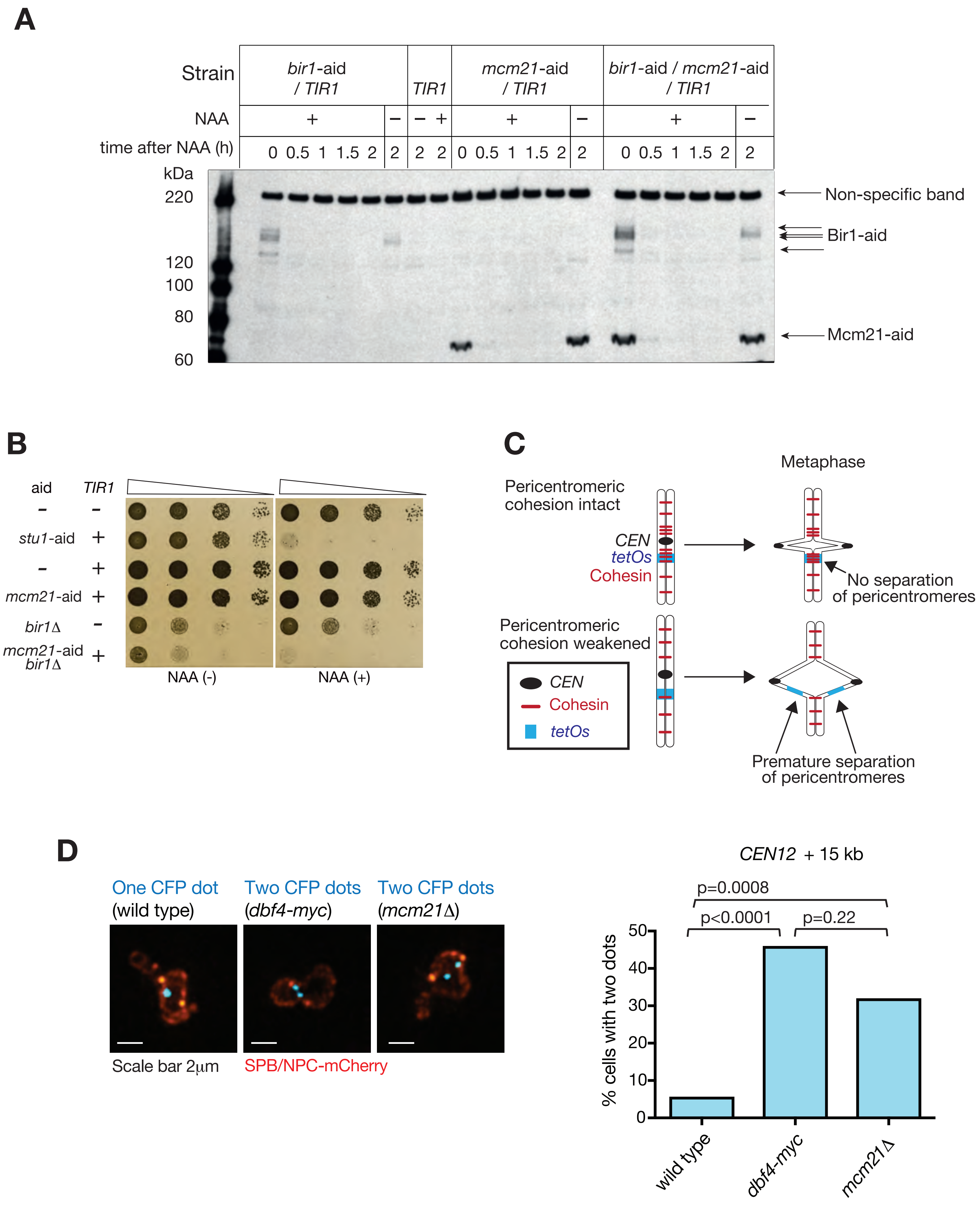

(Figure S1, legend on next page)

Figure S1. Evaluation of protein depletion, synthetic growth defects and peri-centromere cohesion. Related to Figure 1.

A. Bir1-aid and Mcm21-aid are depleted to undetectable levels 30 minutes after addition of NAA. Yeast cells carrying *TIR* with *mcm21-aid* and *bir1-aid* individually and in combination were collected every 0.5 h after addition of 2 mM NAA (or 2 h without NAA, as a control) as indicated. Proteins were separated by the SDS-PAGE (NuPAGE 3-8 % Tris-Acetate gel), blotted and probed with an anti-AID tag antibody. The bands corresponding to Bir1-aid and Mcm21-aid are indicated on the right. The non-specific band of ~220 kDa served as a loading control.

B. Bir1 deletion shows synthetic growth defects when combined with Mcm21 depletion. Yeast cells with *mcm21-aid TIR* and *bir1*Δ individually and in combination, were serially diluted (10 times dilution each), spotted on plates and incubated for 2 days in the presence (right) and absence (left) of NAA. Cells without AID tag or with *stu1-aid* were analysed in the same way as controls.

C. Diagram illustrates that sister *tetO*s at peri-centromeres (e.g. at 15 kb from *CEN12*, see **D**) shows frequent separation during metaphase when peri-centromere cohesion is weakened.

D. *dbf4-myc* shows a defect in peri-centromere cohesion to a similar extent to (or marginally greater than) *mcm21* deletion. *DBF4*$^{+}$ *MCM21*$^{+}$ (wild-type, T10141), *dbf4-myc* (T10142) and *mcm21*Δ (T13365) cells with *tetO*s at 15kb from *CEN12*, *TetR-3×CFP*, *MET3* promoter-*CDC20*, *SPC42-4×mCherry* and *NIC96-4×mCherry* were cultured in methionine drop-out medium, arrested in G1 with α-factor treatment and released into YPAD plus 2 mM methionine, leading to metaphase arrest (due to Cdc20 depletion). At 2 hours after the release, microscopy images were acquired. Spc42 and Nic96 are components of the spindle pole body (SPB) and the nuclear pore complex (NPC), respectively, and SPB signals were much brighter than NPC signals. Images show representative examples where sister *tetO*s are, and are not, separated (two and one CFP dots, respectively). Graphs show % of sister *tetO*s separation during metaphase. n=50–52 for each strain; *p*-values were obtained using Fisher's exact test.

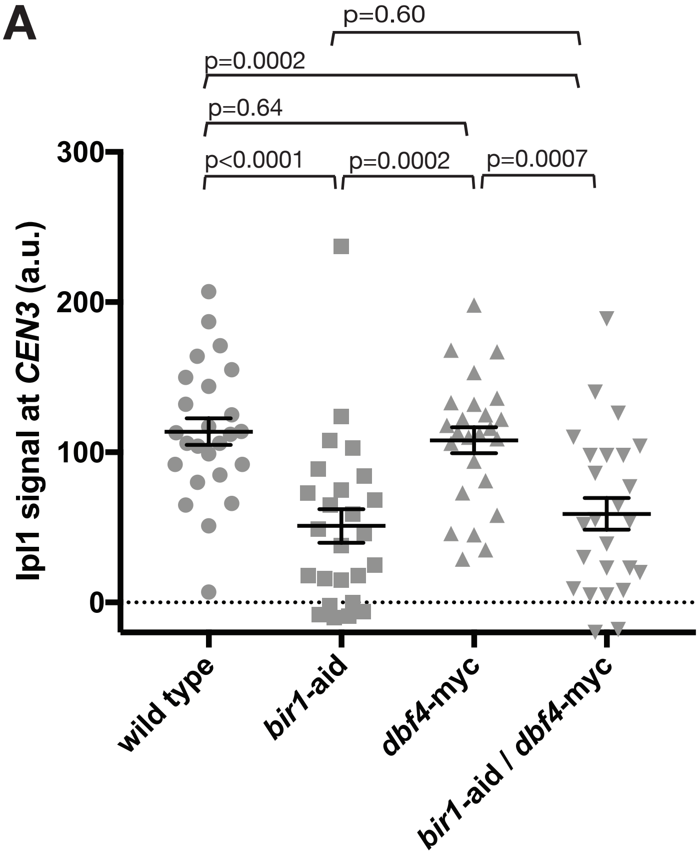

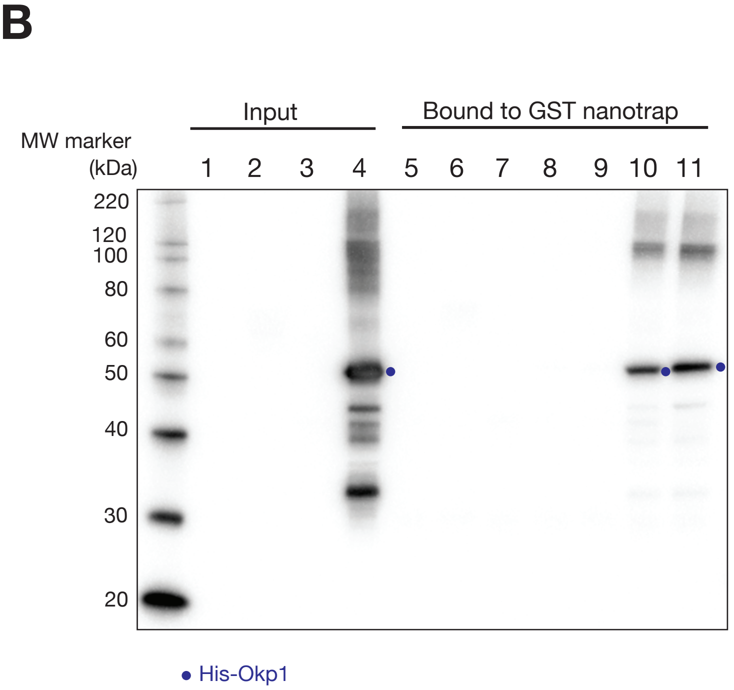

C

	INPUT						PULL-DOWN					
	Lane 2		Lane 3		Lane 4		Lane 9		Lane 10		Lane 11	
	Score	Coverage	Score	Coverage	Score	Coverage	Score	Coverage	Score	Coverage	Score	Coverage
**GST**	**42,125**	93%	**22,124**	92%	3,468	70%	**25,120**	91%	**84,819**	95%	**43,334**	90%
**Sli15 (1-401)**	**79,724**	90%	4,174	65%	3,286	67%	7,308	67%	**141,988**	88%	8,815	70%
**Sli15 (393-698)**	404	45%	**29,749**	89%	590	59%	280	24%	1,030	66%	**37,360**	91%
**Hisx6-Okp1**	63	7%	489	29%	**30,034**	79%	1439	47%	**19,069**	73%	**17,879**	73%
**Ame1**	0	0%	0	0%	**5,977**	72%	200	19%	**3,059**	61%	**3,628**	72%
**Mcm21**	0	0%	90	7%	**6,889**	68%	306	30%	**7,528**	72%	**7,237**	72%
**Ctf19**	0	0%	0	0%	128	14%	0	0%	23	4%	27	4%

(Figure S2, legend on next page)

Figure S2. Effect of *dbf4-myc* on Ipl1 localization at centromeres, and supplemental data for GST pull-down assay. Related to Figure 2.

A. *dbf4-myc* does not affect Ipl1 localization at centromeres in the presence and absence of Bir1. *DBF4*$^{+}$ *BIR1*$^{+}$ (wild-type, T12858), *bir1-aid* (T12860) *dbf4-myc* (T13385) and *bir1-aid dbf4-myc* (T13386) cells with *IPL1-GFP*, *TIR*, *GAL1-10* promoter-*CEN3-tetOs*, *TetR-3×CFP*, *mCherry-TUB1* and *MET3* promoter-*CDC20* were treated and analysed as in Figure 2B and C. Ipl1 signals were quantified at *CEN3* in n=25 cells for each strain. Bars show means and SEMs. *p*-values were obtained using *t*-test.

B. The western blot with an anti-His antibody in Figure 2E is shown in whole.

C. Mass spectrometry analyses of GST pull-down assay. Gel pieces comprising the area between 33 and 75 kDa were excised from Coomassie stained gel in Figure 2E and subjected to mass spectrometry analysis. The data were analyzed with Mascot 2.4.0. The table shows results from lane 2 [Input: GST-Sli15 (1-401)], lane 3 [Input: GST-Sli15 (393-698)], lane 4 [Input: COMA], lane 9 [Pull-down: GST alone + COMA], lane 10 [Pull-down: GST-Sli15 (1-401) + COMA] and lane 11 [Pull-down: GST-Sli15 (393-698) + COMA]. ‘Score’ represents the probability that the observed match is not a random event [$-10\times LOG_{10}(P)$, with P = probability that match is random]. ‘Coverage’ represents the percentage of the protein sequence that is covered by the detected and matched peptides. Scores above the following threshold are indicated in bold: Dependent on background signals, the threshold was set to Score = 10,000 for GST alone, GST-Sli15 (1-401), GST-Sli15 (393-698), and Score = 2,000 for His-Okp1, Ame1, Mcm21 and Ctf19. As most samples contained GST either alone or as a fusion protein, GST was detected with high scores throughout all samples. Lanes 10 and 11 showed a higher abundance of COMA proteins than lane 9, indicating their physical interaction with both GST-Sli15 (1-401) and GST-Sli15 (393-698). Whereas Okp1, Ame1 and Mcm21 were detected with high scores, the score for Ctf19 was reproducibly low. Generally, Ctf19 was hard to detect in mass spectrometry even if it was recovered or expressed in high quantity (e.g. after immuno-precipitation of HA-tagged Ctf19) – perhaps its peptide properties did not allow detection in high sensitivity with mass spectrometry.

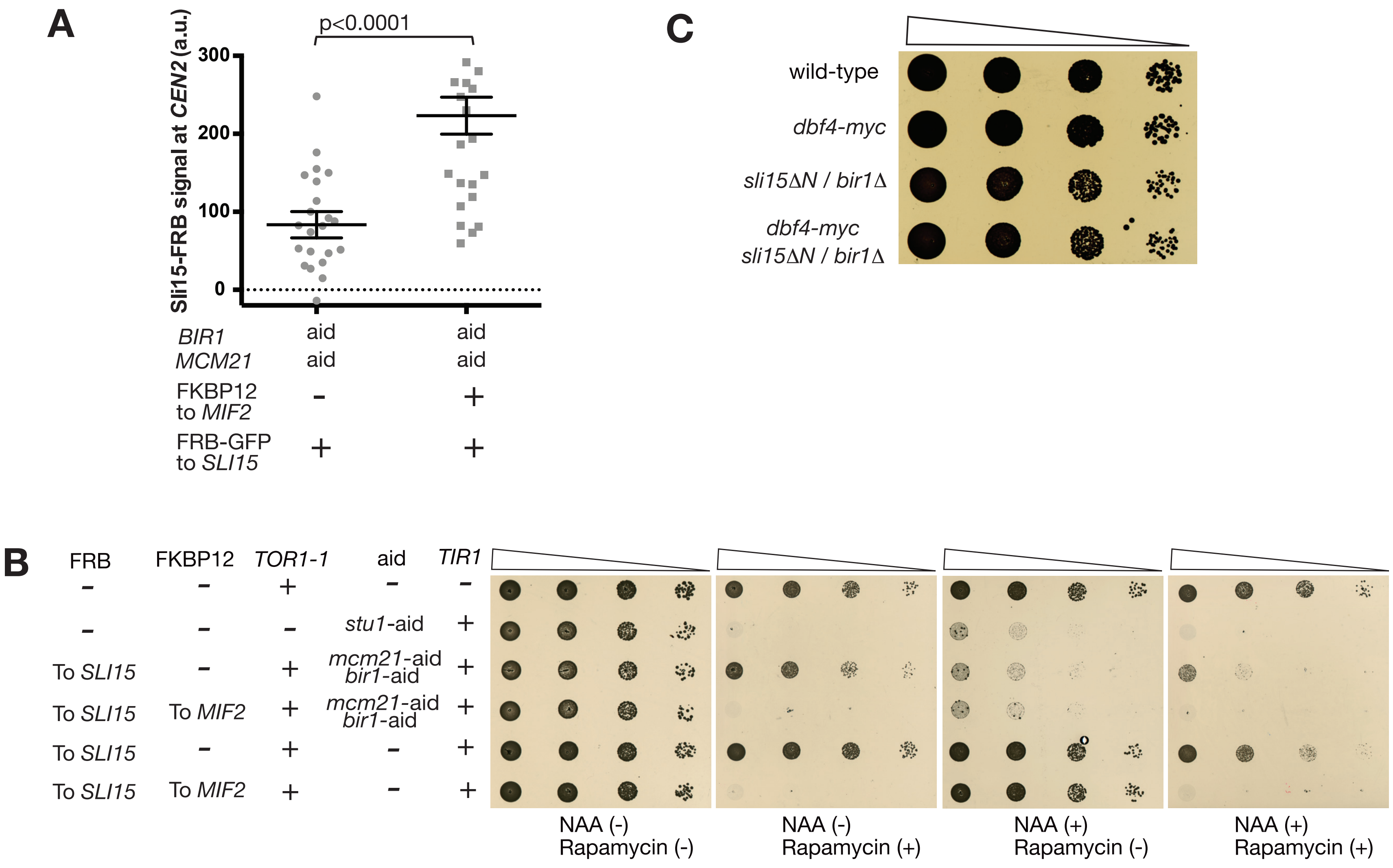

(Figure S3, legend on next page)

Figure S3. Supplemental data for Sli15 tethering to inner kinetochores, and effects of *dbf4-myc* on growth of *bir1*Δ *sli15*ΔN cells. Related to Figure 3 and 4.

A. FKBP12-fused Mif2 recruits FRB-fused Sli15 to the vicinity of *CEN2*. In the experiments shown in Figure 3B, Sli15-FRB signals were quantified at *CEN2* in *bir1-aid mcm21-aid* cells carrying *MIF2* with or without fusion to *FKBP12* (T13444 and T13440, respectively), 30 min after addition of rapamycin. After Z sections were projected to 2D images, Sli15-FRB-GFP signals (colocalising at *CEN2*) were quantified in the area of 2x2 pixels at their maximum intensity. When sister *CEN2* showed separation, Sli15-FRB-GFP signals were averaged between sisters in individual cells. Background was subtracted in every measurement. Bars show means and SEMs. *p*-values were obtained using *t*-test.

B. Tethering Sli15-FRB to Mif2-FKBP12 makes cells inviable. Yeast cells carrying indicated constructs and mutants (the third to sixth strains from the top were the strains used in Figure 3B) were serially diluted (10 time dilution each), spotted on methionine-dropout media with/without NAA and with/without rapamycin (as indicated), and incubated for 3 days. *TOR1-1* cells (T9303) and *stu1*-aid /*TIR* (T10133) were used as controls. We speculate the reason for the lethality of tethering Sli15-FRB to Mif2-FKBP12 as follows: It is known that Ipl1–Sli15 normally leaves centromeres and re-locates to the central spindle during anaphase. We suspect that the presence of Ipl1–Sli15 at the centromere/inner kinetochore during anaphase could cause lethality by disrupting kinetochore–microtubule interaction (since tension is weakened) [S1], reengaging cells to the spindle-assembly checkpoint [S2] and/or delaying the spindle disassembly [S3].

C. *dbf4-myc* does not affect growth of cells with *bir1*Δ *sli15*ΔN. Yeast cells with *bir1*Δ *sli15*ΔN with *DBF4*$^{+}$ or *dbf4-myc*, were serially diluted (10 times dilution each), spotted on plates and incubated for 2 days.

Name	Genotype
PJ69-4A	*MAT* a *trp1-901 leu2-3,112 ura3-52 his3-200 gal4Δ gal80Δ LYS2::GAL1-HIS3 GAL2-ADE2 met2::GAL7-lacZ*
T7107	*MAT* a *ade2-1 trp1-1 leu2-3,112, his311, 15 ura3 can 1-100*
T9030	*MAT* a *ura3::PADH1-OsTIR1-9Myc::URA3*
T9303	*MAT* α *TOR1-1 fpr1Δ::natMX4*
T10133	*MAT* α *ura3::pADH1-OSTIR1-2-9Myc::URA3 stu1-5-aid::clonNAT*
T10141	*MAT* a *cdc20::MET-CDC20::TRP1 ade1::tetR-3CFP::HPH1 SPC42-4mCherry::natMX6 NIC96-4mCherry::natMX6 tetO224 (LEU2) is integrated at +15-kb from CEN12*
T10142	*MAT* a *DBF4-9myc::hphNT1 cdc20::MET-CDC20::TRP1 ade1::tetR-3CFP::HPH1 SPC42-4mCherry::natMX6 NIC96-4mCherry::natMX6 tetO224 (LEU2) is integrated at +15-kb from CEN12*
T12064	*MAT* a *sli15ΔNT* (*Δ2-228)*
T12066	*MAT* a *sli15ΔNT* (*Δ*2-228) *bir1Δ::K.lLEU2*
T12099	*MAT* α *bir1Δ::K.l.LEU2*
T12229	*MAT* a *IPL1-yEGFP::SpHIS5 sl15ΔNT* (*Δ*2-228) *bir1Δ::K.lLEU2 cen15::PGAL-CEN3-tetO2x112::URA3 ade1::tetR-3CFP::HPH1 trp1::PTEF1-mcherry-TUB1::TRP1 cdc20::PMET-CDC20::TRP1*
T12248	*MAT* a *IPL1-yEGFP::SpHIS5 cen15::PGAL-CEN3-tetO2x112::URA3 ade1::tetR-3CFP::HPH1 trp1::PTEF1-mcherry-TUB1::TRP1 cdc20::PMET-CDC20::TRP1*
T12401	*MAT* a *ura3::PADH1-OsTIR1-9Myc::URA3 bir1-aid::clonNAT*
T12410	*MAT* a *ura3::PADH1-OsTIR1-9Myc::URA3 mcm21-aid::clonNAT*
T12466	*MAT* a *sl15ΔNT* (*Δ*2-228) *bir1Δ::K.lLEU2 ura3*::PADH1-OsTIR1-9Myc::*URA3 mcm21*-aid:*clonNAT*
T12467	*MAT* a *sl15ΔNT* (*Δ2-228) ura3::PADH1-OsTIR1-9Myc::URA3 mcm21-aid::clonNAT*
T12613	*MAT* α *bir1Δ::K.l.LEU2 ura3*::*PADH1-OsTIR1-9Myc::URA3 mcm21-aid:clonNAT*
T12614	*MAT* a *bir1-aid*::*clonNAT mcm21-aid*::*clonNAT ura3*::PADH1-*OsTIR1-9Myc::URA3*
T12697	*MAT* a *mcm21-aid::clonNAT ura3::PADH1-OsTIR1-9Myc::URA3 tetO224 (LEU2) is integrated at 0.6-kb from CEN2 ade1::tetR-3CFP::HPH1 SPC42-4mCherry::natMX6*
T12698	*MAT* a *bir1-aid::clonNAT ura3::PADH1-OsTIR1-9Myc::URA3 tetO224 (LEU2) is integrated at 0.6-kb from CEN2 ade1::tetR-3CFP::HPH1 SPC42-4mCherry::natMX6*
T12704	*MAT* a *ura3::PADH1-OsTIR1-9Myc::URA3 tetO224 (LEU2) is integrated at 0.6-kb from CEN2 ade1::tetR-3CFP::HPH1 SPC42-4mCherry::natMX6*
T12714	*MAT* a *bir1*-aid::*clonNAT mcm21*-aid::*clonNAT ura3*::PADH1 *OsTIR1-9Myc::URA3 tetO224 (LEU2) is integrated at 0.6-kb from CEN2 ade1::tetR-3CFP::HPH1 SPC42-4mCherry::natMX6*
T12738	*MAT* a *IPL1-yEGFP::SpHIS5 cen15::PGAL-CEN3-tetO2x112::URA3 ade1::tetR-3CFP::HPH1 trp1::PTEF1-mcherry-TUB1::TRP1 cdc20::PMET-CDC20::TRP1 ura3::PADH1-OsTIR1-9Myc::URA3 mcm21-aid::clonNAT*
T12739	*MAT* a *IPL1-yEGFP::SpHIS5 sl15ΔNT* (*Δ2-228) bir1Δ::K.l.LEU2 cen15::PGAL-CEN3-tetO2x112::URA3 ade1::tetR-3CFP::HPH1 trp1::PTEF1-mcherry-TUB1::TRP1 cdc20::PMET-CDC20::TRP1 ura3::PADH1-OsTIR1-9Myc::URA3 mcm21-aid::clonNAT*
T12809	*MAT* a *DBF4-9Myc::hphNT1 ura3::PADH1-OsTIR1-9Myc::URA3*
T12810	*MAT* alpha *DBF4-9Myc::hphNT1 ura3::PADH1-OsTIR1-9Myc::URA3 bir1-aid::clonNAT*
T12811	*MAT* a *DBF4*-9Myc::*hphNT1 sl15ΔNT* (*Δ*2-228) *bir1Δ::K.lLEU2*
T12858	*MAT* a *IPL1-yEGFP::SpHIS5 cen15::PGAL-CEN3 tetO2x112::URA3 ade1::tetR-3CFP::HPH1 trp1::PTEF1-mcherry-TUB1::TRP1 cdc20::PMET-CDC20::TRP1 ura3::PADH1-OsTIR1-9Myc::URA3*
T12859	*MAT* a *IPL1-yEGFP::SpHIS5 cen15::PGAL-CEN3 tetO2x112::URA3 ade1::tetR-*

	3CFP::HPH1 trp1::PTEF1-mcherry-TUB1::TRP1 cdc20::PMET-CDC20::TRP1 ura3::PADH1-OsTIR1-9Myc::URA3 mcm21-aid::clonNAT
T12860	*MAT* a *IPL1-yEGFP::SpHIS5 cen15::PGAL-CEN3-tetO2x112::URA3 ade1::tetR-3CFP::HPH1 trp1::PTEF1-mcherry-TUB1::TRP1 cdc20::PMET-CDC20::TRP1 ura3::PADH1-OsTIR1-9Myc::URA3 bir1-aid::clonNAT*
T12861	*MAT* a *IPL1-yEGFP::SpHIS5 cen15::PGAL-CEN3-tetO2x112::URA3 ade1::tetR-3CFP::HPH1 trp1::PTEF1-mcherry-TUB1::TRP1 cdc20::PMET-CDC20::TRP1 ura3::PADH1-OsTIR1-9Myc::URA3 mcm21-aid::clonNAT bir1*-aid::*clonNAT*
T13199	*MAT* a *TOR1-1 fpr1Δ::K.l. LEU2 SLI15-FRB-GFP::KanMX6 cen15::PGAL-CEN3-tetO2x112::URA3 ade1::tetR-3CFP::HPH1 trp1::PTEF1-mcherry-TUB1::TRP1 cdc20::PMET-CDC20::TRP1 ura3::PADH1-OsTIR1-9Myc::URA3*
T13200	*MAT* a *TOR1-1 fpr1Δ::K.l. LEU2 MIF2-2xFKBP12::TRP1 SLI15-FRB-GFP::KanMX6 cen15::PGAL-CEN3-tetO2x112::URA3 ade1::tetR-3CFP::HPH1 trp1::PTEF1-mcherry-TUB1::TRP1 cdc20::PMET-CDC20::TRP1 ura3::PADH1-OsTIR1-9Myc::URA3*
T13201	*MAT* a *TOR1-1 fpr1Δ::K.l. LEU2 SLI15-FRB-GFP::KanMX6 cen15::PGAL-CEN3-tetO2x112::URA3 ade1::tetR-3CFP::HPH1 trp1::PTEF1-mcherry-TUB1::TRP1 cdc20::PMET-CDC20::TRP1 ura3::PADH1-OsTIR1-9Myc::URA3 bir1*-aid*::clonNAT*
T13202	*MAT* a *TOR1-1 fpr1Δ::K.l. LEU2 MIF2-2xFKBP12::TRP1 SLI15-FRB-GFP::KanMX6 cen15::PGAL-CEN3-tetO2x112::URA3 ade1::tetR-3CFP::HPH1 trp1::PTEF1-mcherry-TUB1::TRP1 cdc20::PMET-CDC20::TRP1 ura3::PADH1-OsTIR1-9Myc::URA3 bir1-aid::clonNAT*
T13365	*MAT* a *cdc20::MET-CDC20::TRP1 ade1::tetR-3CFP*::HPH1 mcm21Δ::KanMX4 SPC42-4mCherry::natMX6 NIC96-4mCherry::natMX6 tetO224 (LEU2) is integrated at +15-kb from CEN12*
T13385	*MAT* a *DBF4-9Myc::kanMX4 IPL1-yEGFP::SpHIS5 cen15::PGAL-CEN3-tetO2x112::URA3 ade1::tetR-3CFP::HPH1 trp1::PTEF1-mcherry-TUB1::TRP1 cdc20::PMET-CDC20::TRP1 ura3::PADH1-OsTIR1-9Myc::URA3*
T13386	*MAT* a *DBF4-9Myc::kanMX4 IPL1-yEGFP::SpHIS5 cen15::PGAL-CEN3-tetO2x112::URA3 ade1::tetR-3CFP::HPH1 trp1::PTEF1-mcherry-TUB1::TRP1 cdc20::PMET-CDC20::TRP1 ura3::PADH1-OsTIR1-9Myc::URA3 bir1-aid::clonNAT*
T13438	*MAT* a *TOR1-1 fpr1Δ::K.l. LEU2 SLI15-FRB-GFP::KanMX6 ura3::PADH1-OsTIR1-9Myc::URA3 tetO224 (LEU2) is integrated at 0.6-kb from CEN2 ade1::tetR-3CFP::HPH1 SPC42-4mCherry::natMX6 cdc20::MET-CDC20::TRP1*
T13440	*MAT* a *TOR1-1 fpr1Δ::K.l. LEU2 bir1-aid::clonNAT mcm21-aid::clonNAT SLI15-FRB-GFP::KanMX6 ura3::PADH1-OsTIR1-9Myc::URA3 tetO224 (LEU2) is integrated at 0.6-kb from CEN2 ade1*::tetR-3CFP::HPH1 *SPC42-4mCherry::natMX6 cdc20::MET-CDC20::TRP1*
T13441	*MAT* a *TOR1-1 fpr1Δ::K.l. LEU2 MIF2-2xFKBP12::TRP1 SLI15-FRB-GFP::KanMX6 ura3::PADH1-OsTIR1-9Myc::URA3 tetO224 (LEU2) is integrated at 0.6-kb from CEN2 ade1::tetR-3CFP::HPH1 SPC42-4mCherry::natMX6 cdc20::MET-CDC20::TRP1*
T13444	*MAT* a *TOR1-1 fpr1::K.l. LEU2 MIF2-2xFKBP12::TRP1 bir1-aid::clonNAT mcm21-aid::clonNAT SLI15-FRB-GFP::KanMX6 ura3::PADH1-OsTIR1-9Myc::URA3 tetO224 (LEU2) is integrated at 0.6-kb from CEN2 ade1::tetR-3CFP::HPH1 SPC42-4mCherry::natMX6 cdc20::MET-CDC20::TRP1*

Table S1. The genotypes of budding yeast (*Saccharomyces cerevisiae*) strains used in this study (Related to STAR Methods).

The background of all yeast strains is W303 except for PJ69-4A.

Supplemental References

S1. Tanaka, T.U., Rachidi, N., Janke, C., Pereira, G., Galova, M., Schiebel, E., Stark, M.J., and Nasmyth, K. (2002). Evidence that the Ipl1-Sli15 (Aurora kinase-INCENP) complex promotes chromosome bi-orientation by altering kinetochore-spindle pole connections. Cell *108*, 317-329.

S2. Biggins, S., and Murray, A.W. (2001). The budding yeast protein kinase Ipl1/Aurora allows the absence of tension to activate the spindle checkpoint. Genes & development *15*, 3118-3129.

S3. Buvelot, S., Tatsutani, S.Y., Vermaak, D., and Biggins, S. (2003). The budding yeast Ipl1/Aurora protein kinase regulates mitotic spindle disassembly. The Journal of cell biology *160*, 329-339.
